# Histone H2B Deacylation Selectivity: Exploring Chromatin’s Dark Matter with an Engineered Sortase

**DOI:** 10.1021/jacs.1c13555

**Published:** 2022-02-17

**Authors:** Zhipeng A. Wang, Samuel D. Whedon, Mingxuan Wu, Siyu Wang, Edward A. Brown, Ananya Anmangandla, Liam Regan, Kwangwoon Lee, Jianfeng Du, Jun Young Hong, Louise Fairall, Taylor Kay, Hening Lin, Yingming Zhao, John W. R. Schwabe, Philip A. Cole

**Affiliations:** † Division of Genetics, Department of Medicine, Brigham and Women’s Hospital, Boston, Massachusetts 02115, United States; ‡ Department of Biological Chemistry and Molecular Pharmcology, Harvard Medical School, Boston, Massachusetts 02115, United States; § Leicester Institute of Structural and Chemical Biology, Department of Molecular and Cell Biology, University of Leicester, Leicester, LE1 7RH, United Kingdom; ⊥ Howard Hughes Medical Institute; Department of Chemistry and Chemical Biology, Cornell University, Ithaca, New York 14853, United States; ∇ The Ben May Department for Cancer Research, Chicago, Illinois 60637, United States

## Abstract

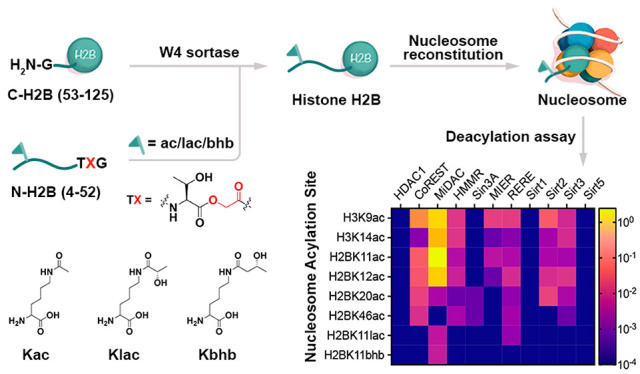

We describe a new method to produce histone H2B by semisynthesis with an engineered sortase transpeptidase. N-Terminal tail site-specifically modified acetylated, lactylated, and β-hydroxybutyrylated histone H2Bs were incorporated into nucleosomes and investigated as substrates of histone deacetylase (HDAC) complexes and sirtuins. A wide range of rates and site-specificities were observed by these enzyme forms suggesting distinct biological roles in regulating chromatin structure and epigenetics.

The writing, erasing, and reading of post-translational modifications (PTMs) on histone tails are critical for regulating chromatin structure and gene expression in healthy and disease states.^1^ Chromatin is comprised of nucleosomes, octameric assemblies of pairs of histones H2A, H2B, H3, and H4 wrapped by ∼146 base pairs of double-stranded DNA.^2^ Extensive efforts to analyze the functions and enzymatic regulation of PTMs on histones H3^3−5^ and H4^6−8^ tails in the context of nucleosomes have benefited from site-specific incorporation of PTMs from chemical biology approaches.^9−11^ By comparison, histone H2B N-terminal PTMs have been understudied.^12^ Recent work, however, has revealed highly dynamic Lys acetylation (Kac) sites on the tail of histone H2B upon acute inhibition of p300/CBP.^13^ H2BK11ac and K12ac, in particular, are among sites with the shortest cellular half-lives (<15 min), while H2BK20ac has a relatively longer cellular half-life (∼30 min) after p300/CBP inhibition. However, the deacetylases responsible for regulating these sites are unknown. By contrast, H2BK46ac, part of the histone H2B core region, appears unaffected by p300/CBP inhibition.^13^ Histones have also been shown to undergo metabolic state-dependent Lys acylation including lactylation (Klac)^14^ and β-hydroxybutyrylation (Kbhb)^15^ on human and mouse chromatin, particularly in disease models,^16^ although with unclear function and enzymatic regulation.^17^

There are two major types of Lys deacylases in mammalian cells, Zn metallohydrolase enzymes known as HDACs, and NAD-dependent deacylases known as the sirtuins.^18^ HDACs like HDAC1 have been identified in a variety of multiprotein complexes including CoREST,^19^ MiDAC,^20^ HMMR (NuRD deacetylase module bound to MBD2),^21^ Sin3A,^22^ MIER,^23^ and RERE.^24^ Each complex is thought to have distinct biological functions, although differences in deacylase activity and site-specificity are uncertain. Understanding the mechanisms of HDAC and sirtuin substrate recognition depends on access to homogeneously acylated protein and nucleosome substrates. Here, we report the scarless semisynthesis of site-specifically modified, full-length histone H2B with an engineered sortase, and the use of these synthetic substrates in unraveling HDAC1 complex and sirtuin selectivity toward acylated nucleosome substrates (Figure 1a,b).

**Figure 1:**
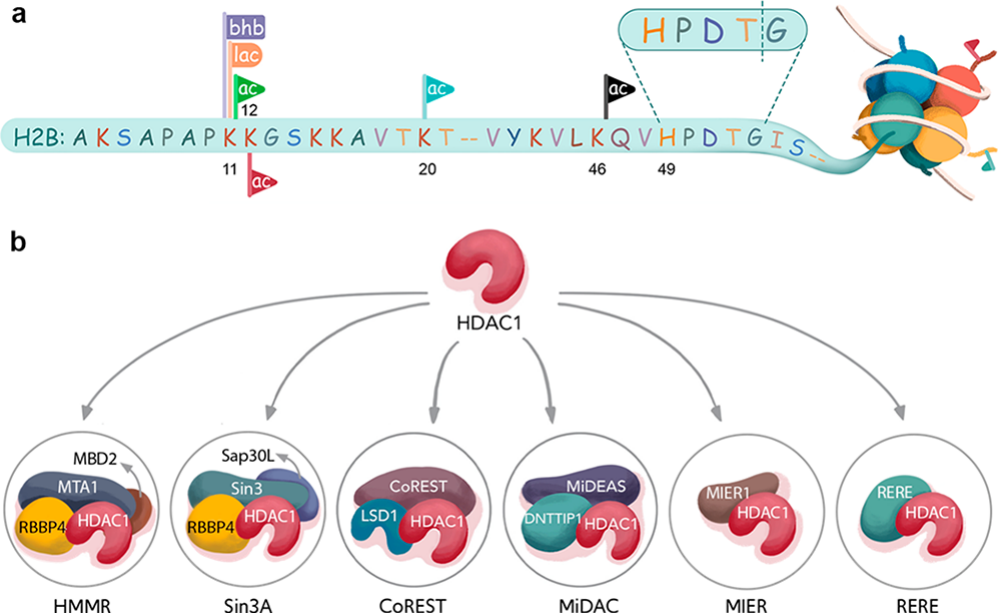
H2B acylations and HDAC complexes. (a) Nucleosome depicting histone H2B acylations and acylation sites, as well as the W4 recognition sequence. (b) HDAC1 complexes studied here.

Standard sortase A recognizes the peptide motif LPXTG, where X is any amino acid, and catalyzes transamidation of the LPXT fragment onto an N-terminal G displacing the C-terminal G in the recognition motif.^25^ Prior efforts have employed an engineered variant of sortase, F40,^26^ to generate semisynthetic histone H3 by acting on the motif A_29_PATG_33_. Histone H2B contains a similar sequence, H_49_PDTG_53_, and we therefore sought a sortase variant that could accommodate a His residue in the first position. Using site-directed mutagenesis, we generated four new sortase mutants (W1–W4) designed to enhance catalysis and/or alter substrate selectivity. We tested these with a simplified H2B peptide containing the HPDTG sequence as a model substrate (Figure S2G), and identified W4 as the most active in cleaving the model substrate (Figure S1, Figure S26, Table S1). We then explored W4 as a catalyst for semisynthesis of *X. laevis* full-length histone H2B.^27,28^ Building on prior work in sortase semisynthesis, we prepared a synthetic N-terminal H2B (aa4–52) peptide (N-H2B) with a depsipeptide (ester) linkage between Thr52 and glycolic acid. Prior studies on histone H3 have revealed the depsipeptide linkage can increase the yield of the desired full-length protein.^29^ With minimal optimization, it was observed that W4 sortase can ligate N-H2B peptide and heterologously expressed C-H2B (aa53–125) (using the corresponding human aa numbering) with ∼40% yield, affording the full-length histone H2B (Figure 2a,b, Figure S3).

**Figure 2:**
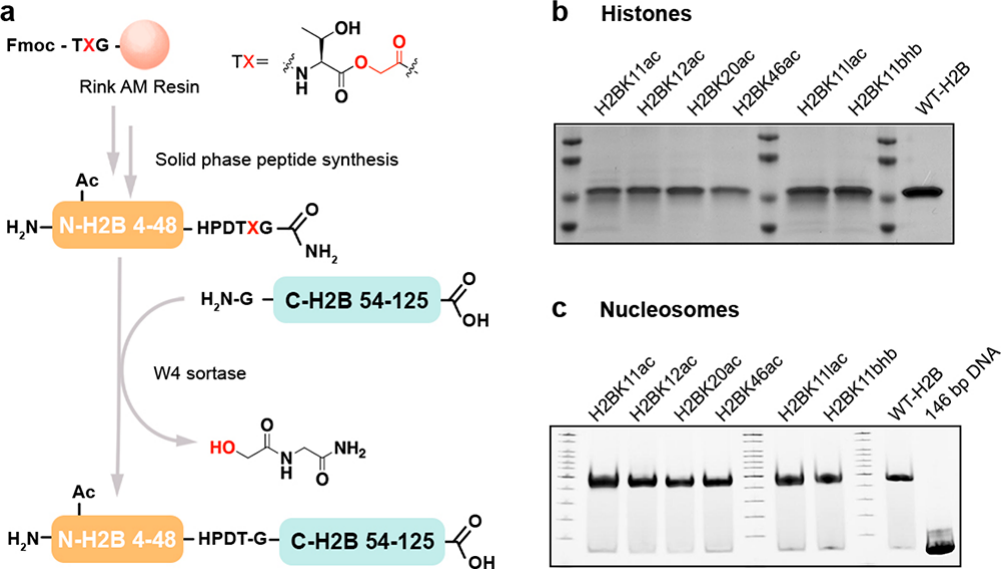
H2B semisynthesis. (a) Scheme for W4 sortase-catalyzed H2B semisynthesis. (b) SDS-PAGE for H2B proteins with Coomassie staining. (c) Native TBE gel for H2B nucleosomes.

Using W4 sortase and the appropriate N-terminally modified peptides, we generated six semisynthetic histone H2Bs including H2BK11ac, H2BK12ac, H2BK20ac, H2B46ac, H2BK11lac, and H2BK11bhb (Figure S4). With H2BK11lac and H2BK11bhb substrates,^30^ we sought to uncover deacylases for these unusual modifications. These semisynthetic H2B histone proteins were purified by RP-HPLC and validated by intact protein mass spectrometry (Figure S3, Figure S5). Each of the modified semisynthetic histones was readily incorporated into histone octamers, and subsequently into nucleosomes with 146 bp 601 Widom dsDNA (Figure 2c).^31^

The modified nucleosomes were assayed with six HDAC1 complexes: CoREST, MiDAC, HMMR, Sin3A, MIER, and RERE as well as free HDAC1,^32^ and four purified sirtuins: Sirt1, Sirt2, Sirt3, and Sirt5 (Figure S6).^33,34^ For comparison, each enzyme/complex was assayed with free semisynthetic modified histone H2B protein. As previously described,^32^ Western blot time course assays with the relevant commercial site-selective acetyl-Lys or acyl-Lys antibodies (Figure S7) were employed (Figure 3). Dilute, free histone H2B appeared to aggregate, particularly under the sirtuin assay conditions, as a function of time so we adjusted the measurement period to short windows to mitigate this complication (Figure S9). We have previously studied the deacetylation kinetics of related complexes using H3K9ac and K14ac nucleosome substrates, as well as free H3K9ac protein, which we have assayed again here to assess consistency with prior studies (Figures S10–16, Figures S20–23). The rates, calculated as velocity/enzyme concentration (V/[E]), are derived from exponential decay curves and shown in Tables S2 and S3, and as heat maps (Figure 4a,b). We have previously characterized the NuRD deacetylase module and find that the HMMR complex displays similar deacetylase activity with H3K9ac nucleosome substrate (Figure S13A).^32^

**Figure 3:**
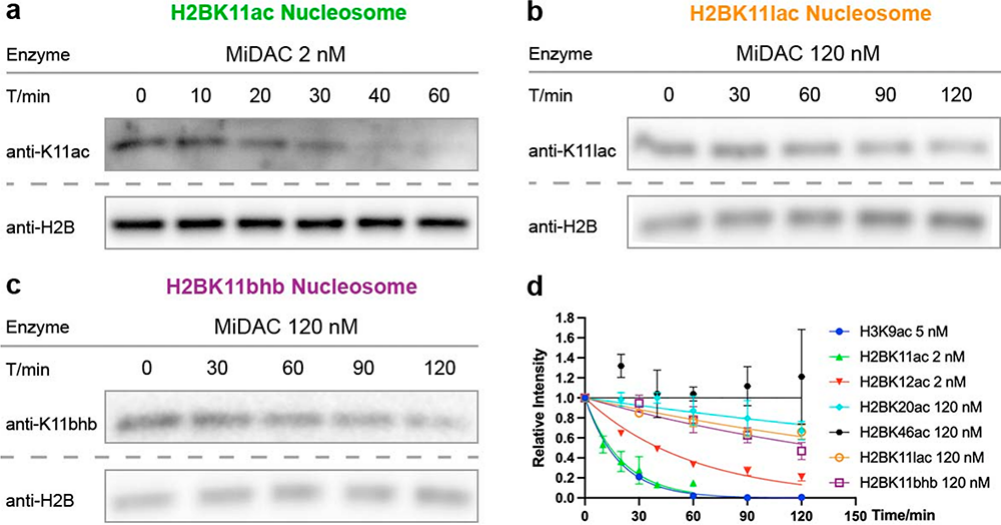
Typical western-blot results for MiDAC assay on H2B nucleosomes installed with (a) H2BK11ac, (b) H2BK11lac, and (c) H2BK11bhb. (d) Exponential decay curve-fitting.

**Figure 4:**
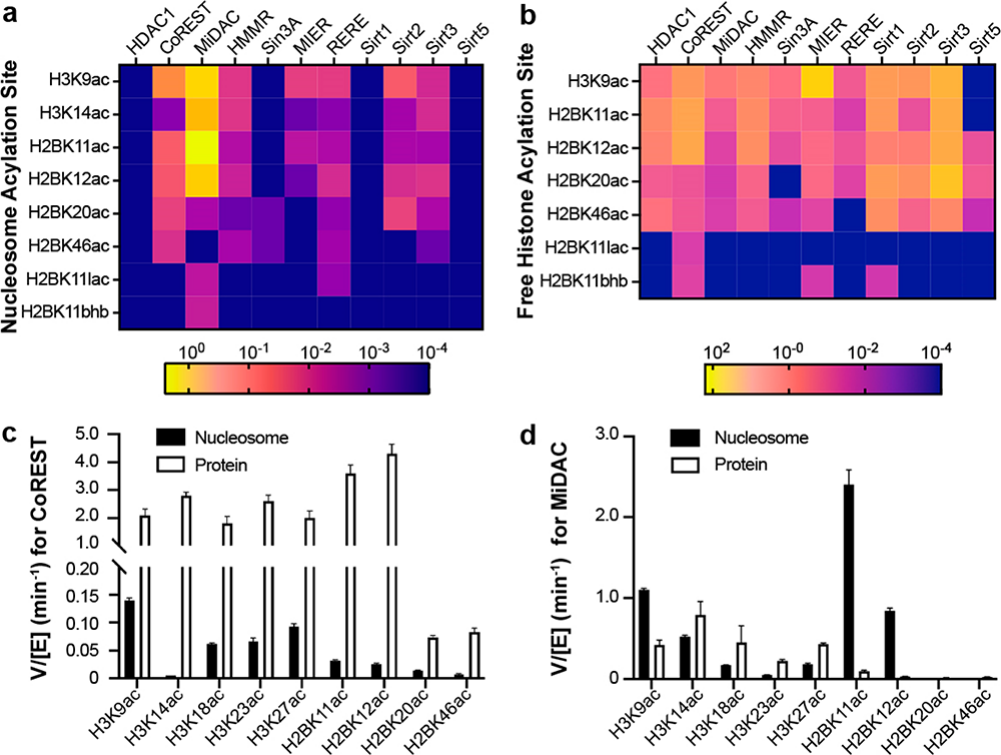
Heatmap for V/[E] (min^–1^) of HDAC1 complexes and sirtuins assays with (a) acylated H3 and H2B nucleosomes, (b) acylated free H3 and H2B proteins. Bar graph of V/[E] (min^–1^) on nucleosome and histone free protein for (c) CoREST, (d) MiDAC. A part of the data for H3K14ac, H3K18ac, H3K23ac, and H3K27ac for both complexes are incorporated from our previous report.^32^

There were several notable findings. As reported previously with acetylated H3 nucleosome substrates,^32^ a wide range of velocities were observed among the HDAC1 complexes with acyl-H2B nucleosome substrates. Striking variation was observed in HDAC1 complexes, with a greater than 2000-fold difference between MiDAC (V/[E] = 2.4 min^–1^) and Sin3A (V/[E] < 0.001 min^–1^) H2BK11ac deacetylation rates. Free HDAC1 was inactive toward any of the H2B nucleosome substrates, consistent with previous observations of acetylated H3 nucleosomes.^32^ Sirtuins showed a narrower dynamic range on modified H2B nucleosome substrates, showing a maximum rate with Sirt2 and H2BK20ac substrate (V/[E] = 0.015 min^–1^).

Most of the HDAC1 complexes, CoREST, HMMR, MIER, and RERE, deacetylate H3K9ac nucleosomes faster than any acetylated H2B nucleosomes. Sirt2 and Sirt3 were the only two sirtuins with detectable activity on nucleosomes and showed similar rates with H3K9ac nucleosomes and H2B acetylated nucleosomes. MiDAC was the only complex found to preferentially deacetylate an H2B site, with ∼2-fold greater activity toward H2BK11ac nucleosome over H3K9ac nucleosome (Figure 4d, Table S2).

In general, nucleosomal H2BK20ac and H2BK46ac were more slowly removed than H2BK11ac and H2BK12ac by both HDAC1 complexes and sirtuins. This observation in nucleosomes is consistent with the proximity of H2BK20 to the DNA backbone, and the position of H2BK46 between α-helices 1 and 2 of the H2B globular domain. This general deacetylase selectivity (H2BK11ac,K12ac > K20ac > K46ac) is consistent with observed cellular acetylation half-lives following p300/CBP inhibition. It is therefore plausible that these enzymes/complexes are principal drivers of cellular deacetylation of p300/CBP histone H2B acetylation sites. In a striking example of site selectivity, the MiDAC complex was found to deacetylate the H2BK11ac nucleosome about 1000-fold faster than the H2BK20ac nucleosome (V/[E] = 0.0022 min^–1^ for H2BK20ac) whereas, for the CoREST and RERE complexes, these rate differences are only ∼2-fold when comparing these two sites.

H2B deacetylation rates were generally slower for nucleosomal substrates than for free histone substrates (Table S2, Table S3) both with HDAC1 complexes and sirtuins (typical examples are CoREST and Sirt3). These results are consistent with deacetylation of modified histone H3 substrates.^32^ Basic histone tails in nucleosomes favorably interact with the acidic phosphate backbone of DNA; thus, it is possible that the faster deacetylation of free histone tails is related to their greater flexibility and accessibility.^35^ In an exception to this trend, MiDAC preferentially processed nucleosomal H2BK11ac (V/[E] = 2.4 min^–1^) and H2BK12ac (V/[E] = 0.84 min^–1^) over the free histone forms (V/[E] = 0.099 min^–1^ and 0.030 min^–1^ for H2BK11ac and H2BK12ac respectively). This may be due to the high affinity of MiDAC for nucleosomes.^20^

HDAC1 complexes have previously been shown to exhibit little selectivity among H3K9ac, K14ac, K18ac, K23ac, and K27ac free histone substrates.^32^ By contrast, both HDAC1 complexes and sirtuins exhibited considerable site selectivity among free H2B acetylation sites. Moreover, some selectivities diverge from nucleosomal substrate selectivities. Free HDAC1 and the HDAC1 complexes examined here prefer H2BK11ac and K12ac over H2BK20ac and K46ac in free histone H2B proteins. As a dramatic example, the CoREST complex deacetylated H2BK11ac (V/[E] = 3.6 min^–1^) and H2BK12ac (V/[E] = 4.3 min^–1^) ∼50-fold faster than H2BK20ac (V/[E] = 0.073 min^–1^) and H2BK46ac (V/[E] = 0.083 min^–1^) (Figure 4c). The sirtuins, however, preferentially deacetylate H2BK20ac relative to the three other sites. This was best exemplified by Sirt2, which deacetylated H2BK20ac (V/[E] = 1.5 min^–1^) ∼25-fold faster than H2BK11ac (V/[E] = 0.059 min^–1^). The range of H2B deacetylation rates reported here is broader than previously observed for H3 substrates, suggesting more intricate molecular recognition of the H2B N-terminal tail by these enzymes. Notably, the MIER complex, characterized here for the first time, shows a greater than 100-fold higher rate of deacetylation of H3K9ac protein (V/[E] = 28 min^–1^) compared to any H2B acetylation site (Figure S17). Furthermore, deacetylation of H3K9ac protein by the MIER complex is over 10-fold faster than any other HDAC1 complex (the second fastest CoREST V/[E] = 2.1 min^–1^) (Table S3).

Consecutive pairs of Lys residues (11–12, 15–16, 20–21, and 23–24) are a distinctive feature of the H2B tail, made more interesting by the observation that all are known to be acylated. Prior characterization of H3 deacetylation revealed a significant role for consecutive Arg-Lys (RK) sequences in directing HDAC1 complex activity. Switching the 8th and 13th residue of nucleosomal H3 (H3K9acR8G and H3K14acG13R) inverted the site selectivity of the CoREST complex, but had little effect on the MiDAC complex.^32^ These observations prompted the question of whether the Lys-Lys (KK) sequence would be similarly discriminatory. HDAC1 complexes and sirtuins showed diverse selectivities for H2BK11ac and K12ac, supporting the role of the amino acid sequence around an acetylation influencing selectivity. Thus, we integrated prior^32^ and current deacetylase kinetic data to visualize trends in amino acid composition surrounding deacetylation sites (Figure S24, Figure S25). We see the preference of HDAC complexes for positively charged R or K flanking the modified K. HDAC complexes further favored small (A, P) or flexible (S, G) residues in the three positions on either side of the modified Lys, with at least one hydrogen bond donor (S, T). A preference for small and or flexible residues could facilitate interaction with HDAC complexes in which folded scaffold domains crowd the active site.^21^

As the local sequence influences molecular recognition of the acylated lysine, so too does the structure of the acylation itself. The rates of deacylation of H2BK11lac and H2BK11bhb were in all cases slower with both nucleosome and histone substrates than the corresponding rates of H2BK11ac removal by both HDAC1 complexes and sirtuins. With nucleosome substrates, only MiDAC and RERE measurably removed either of these acyl-Lys modifications.

MiDAC removed both Klac and Kbhb, whereas RERE was only active against Klac. For free histone H2B substrates, Klac and/or Kbhb deacylase activities were observed with CoREST, MIER, and Sirt1.^17^ To confirm the enzymatic nature of these reactions, the CoREST complex assays were repeated in the presence of the HDAC inhibitor SAHA,^36^ which abolished the deacylase activities (Figure S18). Taken together, the slow deacylation of Klac and/or Kbhb by the HDAC1 complexes and sirtuins suggest that these acyl-Lys groups are nonprimary targets of the HDAC1 complexes or sirtuins. However, lactylation and β-hydroxybutyrylation of Lys residues in proteins, whether enzymatic or nonenzymatic,^37^ also appear to be very slow such that the attachment and removal kinetics appear commensurate.

In summary, we have described a new approach for the semisynthesis of scarless histone H2B using an engineered sortase enzyme. It expands the versatility of sortases in chemical biology^38^ and protein engineering.^39^ This approach allows for the facile incorporation of a range of chemical modifications from the N-terminus to the core region of H2B. With the semisynthetic acyl H2B nucleosomes, we have identified the MiDAC and CoREST complexes as the most robust deacetylases, and detected activity across a range of other HDAC1 complexes and sirtuins. Diverse site selectivities and magnitudes of the deacetylase activity were observed among the complexes and sirtuins with nucleosomal and free histone H2B substrates. The remarkable variation in HDAC1 complex activity, despite sharing an identical catalytic core polypeptide, HDAC1, highlights the importance of the other subunits in controlling deacetylase activities and molecular recognition. This is consistent with putative specific biological roles of different deacetylases and their complexes in different cellular functions and states. We have also found that Klac and Kbhb modifications in histone H2B are susceptible to enzymatic cleavage, albeit at moderate rates. Overall, we believe that these findings provide a framework for elucidating how specific modifications of histone H2B may influence gene regulation and cellular behaviors.

## Supporting Information Available

Supporting figures and detailed experimental procedures (PDF)
