# Supplementary material for: Histone H2B Deacylation Selectivity: Exploring Chromatin’s Dark Matter with an Engineered Sortase
Source: J Am Chem Soc. 2022 Feb 17;144(8):3360–4. doi: 10.1021/jacs.1c13555 (PMC8895396; doi:10.1021/jacs.1c13555)
Supplement: Supplementary file 1 [file ja1c13555_si_001.pdf]

Supporting Information

Histone H2B Deacylation Selectivity: Exploring Chromatin's Dark Matter with an Engineered Sortase

Zhipeng A. Wang,[†,‡,#] Samuel D. Whedon,[†,‡,#] Mingxuan Wu,[†,‡,‖,#] Siyu Wang,[§] Edward A. Brown,[§] Ananya Anmangandla,[^] Liam Regan,[§] Kwangwoon Lee[†,‡], Jianfeng Du,[▿] Jun Young Hong,[^] Louise Fairall,[§] Taylor Kay,[†,‡] Hening Lin,[^] Yingming Zhao,[▿] John W.R. Schwabe,[§,*] and Philip A. Cole.[†,‡,*]

[†] Division of Genetics, Department of Medicine, Brigham and Women's Hospital, Boston, MA, United States, 02115;

[‡] Department of Biological Chemistry and Molecular Pharmacology, Harvard Medical School, Boston, MA, United States, 02115;

[§] Leicester Institute of Structural and Chemical Biology, Department of Molecular and Cell Biology, University of Leicester, Leicester, United Kingdom, LE1 7RH;

[^] Howard Hughes Medical Institute; Department of Chemistry and Chemical Biology, Cornell University, Ithaca, NY, United States. 14853;

[‖] Westlake University, Hangzhou, Zhejiang Province, China, 310024;

[▿] The Ben May Department for Cancer Research, Chicago, IL, United States, 60637;

Table of Contents

1. Design and expression of sortase mutants 3
2. Evaluation of sortase activity with a model peptide 4
3. Synthesis of depsipeptide Fmoc-Thr(OtBu)-glycolic acid (TOG) 4
4. Synthesis of acetylated N-H2B (aa4-52) TOG peptide 5
5. Synthesis of acylated N-H2B (aa4-52) TOG peptide 6
6. Synthesis of H2B model peptides 6
7. Cloning C-H2B (53-125) plasmid 6
8. Expression of histone H2A, H2B, H4, and histone C-H2B 6
9. W4 sortase-catalyzed histone H2B semisynthesis 7
10. Octamer refolding and nucleosome reconstitution 8
11. Expression and purification of HDAC complexes 8
12. Analysis of HDAC deacylation of acylated H2B & H3 nucleosomes and histones 9
13. SAHA inhibition assay of CoREST on H2BK11lac and H2BK11bhb proteins 10
14. Comparison of NuRD and HMMR deacetylation kinetics on H3K9ac nucleosome 10
15. Fluor de Lys assay on commercial HDAC1 10
16. Cloning, expression, and purification of sirtuins 10
17. Fluor de lys assay on commercial Sirt1 10
18. Analysis of sirtuin deacylation of acylated H2B & H3 nucleosomes and histones 11
19. WT-H2B and WT-H3 assay with different concentration of sirtuins 11
20. Enzyme concentration-dependent assay for HDAC1 complexes and sirtuins 11
21. Complex-specific rate-weighted ranking of local sequences 11
22. Figure S1. Representative RP-HPLC chromatogram of model peptide conversion. 13
23. Table S1. Activity of sortase mutants on a model peptide substrate. 14
24. Figure S2. All MS for N-H2B peptides and model peptides. 15
25. Figure S3. Condition optimization of W4 sortase-catalyzed histone H2B semisynthesis 16
26. Figure S4. Representative semi-prep HPLC chromatography for W4 sortase-catalyzed H2B synthesis 17
27. Figure S5. All analytic HPLC chromatography and MALDI-TOF for full-length H2B .. 18
28. Figure S6. SDS-PAGE for All HDAC1 complexes and sirtuins 19
29. Figure S7. Antibody affinity and specificity tests 20
30. Figure S8 Representative TBE gel for nucleosomes before and after treatment with an HDAC complex (Sin3A) or sirtuin (Sirt2) 21
31. Fig. S9. WT-H2B and WT-H3 protein aggregation test in sirtuin reaction buffer 22
32. Figure S10. WB and curve fitting for deacylation by free HDAC1 23
33. Figure S11. WB and curve fitting for deacylation by the CoREST complex 24
34. Figure S12. WB and curve fitting for deacylation by the MiDAC complex 25
35. Figure S13. WB and curve fitting for deacylation by the HMMR complex 26
36. Figure S14. WB and curve fitting for deacylation by the Sin3A complex 27
37. Figure S15. WB and curve fitting for deacylation by the MIER complex 28
38. Figure S16. WB and curve fitting for deacylation by the RERE complex 29
39. Figure S17. Enzyme concentration-dependent assay for MIER 30
40. Figure S18. SAHA inhibition assay for CoREST on H2BK11lac and H2BK11bhb 31
41. Figure S19. Fluor de lys assay for HDAC1 and sirtuin 1 32
42. Figure S20. WB and curve fitting for deacylation by sirtuin 1 33
43. Figure S21. WB and curve fitting for deacylation by sirtuin 2 34

44. Figure S22. WB and curve fitting for deacylation by sirtuin 3 35
45. Figure S23. WB and curve fitting for deacylation by sirtuin 5 36
46. Table S2. V/[E] of all HDAC1 complexes and sirtuins on H2B and H3 nucleosomes 37
47. Table S3. Normalized V/[E] of all HDAC1 complexes and sirtuins on H2B and H3 free proteins 38
48. Figure S24. Rate-weighted sequence features of HDAC complex target sites.................39
49. Figure S25. Rate-weighted sequence features of sirtuin target sites 40
50. Figure S26. W4 sortase mutation sites 41

1. Design and expression of sortase mutants

Sortase mutants were derived from *S. aureus* sortase A (Srt A). The previously reported enhanced sortase pentamutant (eSrt)[1], F40 sortase mutant[2], and calcium-independent sortase mutations (7M[3], and 7Y[4]) were used as starting points. The substrate recognition loop targeted in the development of F40 sortase was found to alter sorting motif preference from LPXTG to APXTG, and thus was further mutated in an attempt to enhance recognition of the sequence HPXTG. DNA encoding S. aureus Srt A (60-206) (from Dirk Schwarzer lab) was cloned into pET21 vector (Ampicillin resistant). Subsequent mutations were accomplished by sequential rounds of quick-change site directed mutagenesis. The PCR products were purified by PCR cleanup kit (Zymo), treated with DpnI (New England Biolabs), and transformed into DH5α *E. coli*. Single colonies were picked and grown over night in 5 mL of Luria Bertani media supplemented with ampicillin (100 µg / mL), then pelleted (5 minutes, 4500 rcf, 4 ˚C). Plasmids were obtained from cell pellets by mini-prep (Plasmid Miniprep Classic, Zymo) and Sanger sequenced.

F40 amino acid sequence (mutations distinguishing F40 from Srt A are underlined)
MQAKPQIPKDKSKVAGYIEIPDADIKEPVYPGPATPEQLNRGVSFAEENESLDDQNISIAGHTFIDR
PNYQFTNLKAAKMGSMVYFKVGNETRKYKMTSIRDVKPQDVGMHLAEKGKDKQLTLITCDDY
NEKTGVWEKRKIFVATEVKLEHHHHHH

W1 amino acid sequence (mutations distinguishing F40 from Srt A are underlined, mutations from eSrt are *italicized*, mutations from 7M are **bolded**)
MQAKPQIPKDKSKVAGYIEIPDADIKEPVYPGPAT*S*EQLNRGVSFA**K**EN**Q**SLDDQNISIAGHTFID
RPNYQFTNLKAAKMGSMVYFKVGNETRKYKMTSIR*N*VKPQDVGMHLAEKGKDKQLTLITCDD
YNE*E*TGVWE*T*RKIFVATEVKLEHHHHHH

W2 amino acid sequence (mutations distinguishing F40 from Srt A are underlined, mutations from eSrt are *italicized*, mutations from 7M are **bolded**, Gly176 is deleted)
MQAKPQIPKDKSKVAGYIEIPDADIKEPVYPGPAT*S*EQLNRGVSFA**K**EN**Q**SLDDQNISIAGHTFID
RPNYQFTNLKAAKMGSMVYFKVGNETRKYKMTSIR*N*VKPQDVMHLAEKGKDKQLTLITCDDY
NE*E*TGVWE*T*RKIFVATEVKLEHHHHHH

W3 amino acid sequence (mutations distinguishing F40 from Srt A are underlined, mutations from eSrt are *italicized*, mutations from 7M are **bolded**, Val166Ile)
MQAKPQIPKDKSKVAGYIEIPDADIKEPVYPGPAT*S*EQLNRGVSFA**K**EN**Q**SLDDQNISIAGHTFID
RPNYQFTNLKAAKMGSMVYFKVGNETRKYKMTSIR*N*VKPQDIGMHLAEKGKDKQLTLITCDDY
NE*E*TGVWE*T*RKIFVATEVKLEHHHHHH

W4 amino acid sequence (mutations distinguishing F40 from Srt A are underlined, mutations from eSrt are *italicized*, mutations from 7Y are **bolded**)

MQAKPQIPKDKSKVAGYIEIPDADIKEPVYPGPAT*S*EQLNRGVSFA**K**EN**Q**SLDDQNISIAGHTFI**G**
RPNYQFTNLKAAKMGSMVYFKVGNETRKYKMTSIR*N*VKPQDVGMHLAEKGKDKQLTLITCDD
LN**R***E*TGVWE*T*RKIFVATEVKLEHHHHHH

All constructs were transformed into BL21-CodonPlus (DE3) RIPL *E. coli* strain. Protein expression began with inoculation of 50 mL of Luria Bertani (LB) media supplemented with 100 mg/L ampicillin with scrapings from a frozen cell stock. The 50 mL culture was grown overnight (12-16 hr) at 37 ˚C with constant shaking (200 rpm). Subsequently 1 L LB cultures with 100 mg/L ampicillin were inoculated with 10 mL of small-scale culture and grown at 37 ˚C, 200 rpm to an OD_{600} of 0.3-0.5, then brought down in temperature to 30 ˚C. At an OD_{600} of 0.5-0.6 overexpression was induced with the addition of IPTG to 0.3 mM. Cells were grown for a further 3 hr at 30 ˚C, 200 rpm. Cultures were pelleted by centrifugation at 5,000 × g, 4 ˚C, and the supernatant removed. Cell pellets were either stored at -80 ˚C, or immediately processed. Cell pellets were resuspended in 5 pellet volumes ice cold lysis buffer (20 mM Tris, pH 7.5, 0.1% Triton X-100) and lysed by three passages through a french pressure cell. Cell pellets stored at -80 C are notably more difficult to lyse in this fashion, as they become viscous upon thawing. Lysates were clarified by centrifugation at 13,000 x g, 4 ˚C, and lysate supernatants were applied to Ni-NTA resin (2.5 mL resin bed volume / 1 L culture) pre-equilibrated with cold lysis buffer. Column binding was achieved by passing supernatant over the resin bed twice by gravity at a moderate drip rate. The resin bed was washed with 10 column volumes of lysis buffer, followed by 20 volumes of wash buffer (20 mM Tris, pH 7.5, 500 mM NaCl), and 10 volumes of lysis buffer supplemented with 5% elution buffer (20 mM Tris, pH 7.5, 400 mM imidazole). Protein was eluted in five fractions of elution buffer, each of 2 column volumes. Elution fractions were checked by SDS-PAGE (15%) and pooled for dialysis. Generally, all 5 fractions were of sufficient purity for pooling. Purified protein was dialyzed three times against 40 volumes of 50 mM Tris, pH 7.5, 150 mM NaCl, 5 mM $CaCl_2$ at 4 ˚C. This protein was then concentrated by spin concentrator (Amicon, 10 kDa MWCO, 4,500 rpm, 4 ˚C) to a final concentration of 1-3 mM by the absorbance at 280 nm measured by nanodrop (Thales).

2. Evaluation of sortase activity with a model peptide

The H2B model peptide KQVHPDTGGK(ivDde) (0.1 mM) and GGGRG (1 mM) (Figure S2G,H) were combined with buffer (20 mM PIPES, pH 7.0, 1 mM DTT) in 500 µL Eppendorf tubes. The reaction was initiated by the addition of sortase (0.1 mM), or, in the case of initial timepoints 10 % trifluoroacetic acid (to a final concentration of 1 %) followed by sortase. Reactions were run for 24 hr in a 37 ˚C incubator to avoid changes in volume arising from evaporation within the Eppendorf tube. At 24 hr reactions were quenched by the addition of 10 % trifluoroacetic acid (to a final concentration of 1 %). Substrate conversion was assessed by HPLC with C18 analytic column (Agilent, Eclipse XDB-C18, 4.6×250 mm) using area under the curve at 214 nm (Star Chromatography Workstation 6, Varian). Peptide species were confirmed by ESI-MS (Q Exactive Orbitrap, Thermo). A species corresponding to the substrate peptide, KQVHPDTGG(ivDde), -18 Da was observed over the course of the reaction, and attributed to reversible intramolecular imine formation. This species was observed to decrease over the course of the reaction along with the starting material. For the purposes of calculating consumption of the peptide starting material the area under the curve for both the peak of the anticipated mass, and the -18 Da peak were used. All measurements were made in at least triplicate (Figure S1, Table S1).

3. Synthesis of depsipeptide Fmoc-Thr(OtBu)-glycolic acid (TOG)

The synthesis of depsipeptide Fmoc-Thr(OtBu)-glycolic acid was carried out as previously reported in a two-step manner.[5] For the first step, Fmoc-Thr(tBu)-OH (40 g, 100 mmol) was dissolved in 150 mL tetrahydrofuran (THF) together with phase-transfer catalyst tetra-n-butylammonium iodide (TBAI, Sigma8221520100) (5 g). After the addition of benzyl 2-bromoactate (Sigma-Aldrich245631-250G) (1.5 eq, 24 mL, 150 mmol), the coupling was initiated by the addition of N,N-Diisopropylethylamine (DIPEA) (1.1 eq, 19 mL, 110 mmol). The reaction mixture was stirred at room temperature and monitored by thin-layer chromatography (TLC) (hexane:ethyl acetate=5:1). After reaction completion (ca. 6 h), the THF was

then removed by rotovap, and the reaction mixture was extracted three times with 300 mL water/ethyl acetate (EtOAc). The organic layer was combined, washed with water and brine, then dried with anhydrous calcium chloride. The crude product was concentrated and applied to silica gel column chromatography with a linear gradient from 10 % EtOAc/hexane to 40 % EtOAc/hexane over 30 min. The final product (~55 g, yield ~98 %) as a light yellow powder was confirmed by ^{1}HNMR: 8-7.5 ppm (13 H, m), 5.7 ppm (1H, d), 5.2 ppm (2H, s), 4.8-4.7 ppm (2H, t), 4.4 ppm (3H, m), 4.3 ppm (2H, m), 1.3 ppm (12H, m).

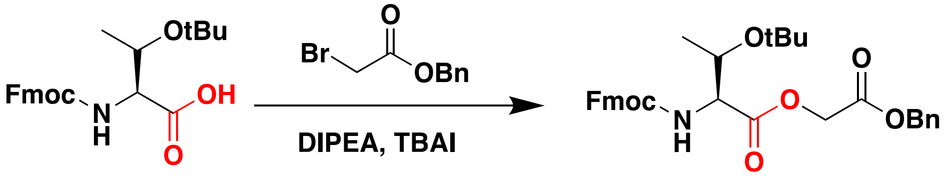

For the second step, the product from the previous step (5.4 g, 10 mmol) was dissolved in a de-gassed solvent mixture of THF/water (v/v 3:1). A catalytic amount of palladium on carbon (Pd/C, Alfa Aesar45924-06) (10-20 %, 540 mg) was added slowly and carefully, followed by repeated degassing. The mixture in a round-bottomed flask was connected to a hydrogen balloon, inserted into rubber septum with a with a long needle. After purging for 1 min, the system was sealed and stirred at room temperature and monitored by TLC (hexane:EtOAc=5:1 to show starting material followed by dichloromethane (DCM)/methanol=9:1 to show product). After reaction completion (ca. 8 h), the THF was largely first removed by rotavap, and the mostly dried reaction mixture was extracted three times with 50 mL water/EtOAc. The organic layer was combined, washed with water and brine, then dried over anhydrous sodium sulfate. The crude product was concentrated and applied to silica gel column chromatography. Pure DCM could elute reaction byproducts, while an isocratic 10% methanol/DCM was used to elute the final product TOG as a white to light yellow powder (~1.2 g, yield ~30 %) confirmed by ^{1}HNMR: 8-7.5 ppm (8H, m), 5.6 ppm (1H, d), 5.0-4.7 ppm (2H, t), 4.4 ppm (3H, m), 4.3 ppm (2H, m), 1.3 ppm (12H, m).

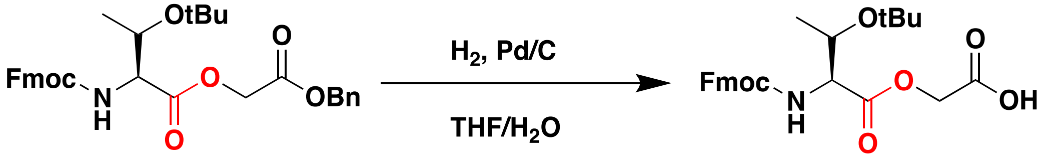

4. Synthesis of acetylated N-H2B (aa4-52) TOG peptide

All the acetylated histone H2B peptides (aa4–52) TOG with site-specific lysine acetylation (Kac) (H2BK11ac, H2BK12ac, H2BK20ac, and H2BK46ac) were synthesized by Fmoc-SPPS with Rink Amide AM resin (EMD Millipore). Syntheses were performed using a Prelude automated peptide synthesizer (Gyros Protein Technologies) using the following deprotection & coupling cycle for a 0.2 mmol scale synthesis: 5 mL of 20% piperidine in DMF was added to the reaction vessel and mixed for 10 min, the vessel was drained, and a second identical deprotection followed; 4 mL of DMF was added to the reaction vessel and mixed for 30 sec, the vessel was drained, and five more washes followed alternating solvent delivery from the top and bottom of the reaction vessel; 0.8 mmol (4 eq) Fmoc-amino acid in 4 mL DMF, and 0.75 mmol (3.75 eq) HATU and 1.6 mmol (8 eq) N-methylmorpholine (NMM) in 4 mL DMF were sequentially added to the reaction vessel and mixed for 90 min, followed one wash with 4 mL DMF (as before), and a second identical coupling. After the coupling of the first Gly, the second amino acid coupled in each case was the depsipeptide TOG, which was hand coupled overnight with 0.4 mmol (2 eq) TOG, 0.36 mmol (1.8 eq) HATU, and 0.8 mmol (4 eq) DIPEA. After all the coupling steps and deprotection steps, the resin was sequentially washed with DMF and dichloromethane (DCM), then dried under vacuum. Cleavage of the peptide from the resin and removal of the side chain protecting groups was accomplished by addition of Reagent B (5 % water, 5 % phenol, 2.5 % triisopropyl silane (TIPS), 87.5 % trifluoroacetic acid (TFA)) to dried resin, followed by 2.5 hr of end-over-end rotating at room temperature was applied to cleave the peptide from the resin and to remove the side chain protecting groups. The crude peptide was concentrated by drying under a stream of dry nitrogen and precipitated with the addition of cold diethyl

ether. The precipitated peptide suspension was centrifuged for 5 min at 3,500 × g, 4 ˚C, and the supernatant was carefully removed. The pellet was washed two more times with cold diethyl ether, removing the supernatant each time, and then dried under nitrogen gas flow for 2 hr. The dry crude peptide was dissolved in 10 % acetonitrile (CH_3CN)-water mixture with 0.05 % TFA, and purified by reverse-phase HPLC over a C8 semi-prep column (Varian Dynamax Microsorb 100, 250×21.4mm), with a linear gradient from 25 % CH_3CN/0.05 % TFA to 55 % CH_3CN/0.05 % TFA over 30 min, at a flow rate of 10 mL/min. Pure fractions were determined by MALDI-TOF MS (Dana Farber Cancer Institute Molecular Biology Core Facilities, 4800 MALDI TOF/TOF, Applied Biosystems/MDS Sciex), combined, frozen and lyophilized to powders (Figure S2A-D).
Sequence: AKSAPAP-**KK**GSKKAVT**K**-TQKKDGKKRR-KTRKESYAIY-VYKVL**K**QVHP-D-TOG-G;
H2BK11ac (4-52) TOG $[M + H]^+$ calculated as m/z 5729.7, observed at m/z 5730.4;
H2BK12ac (4-52) TOG $[M + H]^+$ calculated as m/z 5729.7, observed at m/z 5728.9;
H2BK20ac (4-52) TOG $[M + H]^+$ calculated as m/z 5729.7, observed at m/z 5730.7;
H2BK46ac (4-52) TOG $[M + H]^+$ calculated as m/z 5729.7, observed at m/z 5728.6.

5. Synthesis of acylated N-H2B (aa4-52) TOG peptide

The synthesis of H2BK11alloc (aa4-52) TOG was carried out using the general protocol described above. Alloc deprotection was performed as previously by $Pd(PPh_3)_4$ as reported[6] (*L*)-lactic acid (Sigma L1750-10G) or (*R*)-β-hydroxybutyric acid (Aldrich 54920-1G-F) was conducted manually: carboxylic acid (10 eq) was mixed together with HBTU (9 eq) and DIPEA (20 eq) in 5 mL DMF, and applied to the resin for a 90 min, followed by a second identical coupling. The peptides were then cleaved, purified and characterized as described above (Figure S2E,F).
H2BK11lac (4-52) TOG $[M + H]^+$ calculated as m/z 5760.7, observed at m/z 5760.9;
H2BK11bhb (4-52) TOG $[M + H]^+$ calculated as m/z 5774.7, observed at m/z 5778.6;

6. Synthesis of H2B model peptides

The synthesis and purification of GGGRG was carried out with a similar protocol as described above, starting from Rink Amide AM resin (EMD Millipore). The crude peptide dissolved in water with 0.05 % TFA and purified by RP-HPLC using a C18 preparative column with integral polar functionality (ACE-126-1520, 150 x 21.2 mm, Advance Chromatography Technologies LTD) and an isocratic solvent composition (water with 0.05 % TFA). The synthesis and purification of KQVHPDTGGK(ivDde) was carried out using the general protocol described above, starting from Fmoc-Lys(ivDde)-Wang resin (Nova Biochem, # 856186) (Figure S2G,H).
GGGRG $[M + H]^+$ calculated as m/z 402.4, observe at m/z 402.0;
KQVHPDTGGK(ivDde) $[M + H]^+$ calculated as m/z 1273.2, observed at m/z 1272.7.

7. Cloning C-H2B (53-125) plasmid

Residues 4-52 were excised from a plasmid encoding *Xenopus laevis* histone H2B (pET-H2B) by polymerase chain reaction (PCR) with PfuUltra II Fusion HS DNA polymerase in PfuUltra buffer (Agilent 600670) and the following primers:
H2B4-52Del-F: 5'-TTA AGA AGG AGA TAT ATA TGG GCA TCT CGT CCA AGG CCA T-3'
H2B4-52Del-R: 5'- ATG GCC TTG GAC GAG ATG CCC ATA TAT ATC TCC TTC TTA A-3'
Template plasmid was digested with DpnI (New England Biolabs #R0176L) in CutSmart buffer (10×, New England Biolabs) for 3 hr at 37 ˚C. The DNA product pET-C-H2B (53-125) was purified by PCR cleanup kit (Zymo #11-301C), and transformed into competent DH5α *E. coli*, followed by plating on LB agar plates supplemented with ampicillin (100 µg/mL). Single colonies were selected, grown, and sequenced as described above.

8. Expression of histone H2A, H2B, H4, and histone C-H2B

Wild type (WT) *Xenopus laevis* core histones H2A, H2B, H3, and H4[7], together with H3 (aa34-135, with the first Met cleaved by *E. coli*) (gH3) and H2B (aa53-125, with the first Met cleaved by *E. coli*) (C-H2B)

were expressed and purified as previously reported[8]. Both H3 and gH3 constructs carry a C110A mutation, which is widely used in both nucleosome chemical biology[9] and nucleosome structural studies[10]. The pET-C-H2B plasmid described above was used to transform *E. coli* Rosetta (DE3) pLysS, and plated on LB agar plates supplemented with ampicillin (100 μg/mL). After growing overnight (12-16 h) at 37 ˚C, a single colony was picked to inoculate 1 L LB media supplemented with 100 mg/L ampicillin. The culture was grown at 37 ˚C while shaking at 200 rpm until the OD_{600} was 0.6. Expression of C-H2B was induced with 0.5 mM IPTG at 37 ˚C for 2 hr. Cells were harvested by centrifugation at 4,000 rpm for 15 min and supernatant was removed. The cell pellet was resuspended in histone lysis buffer (50 mM Tris-HCl at pH 7.5, 100 mM NaCl, 1 mM EDTA, 5 mM 2-mercaptoethanol (BME), 0.2 mM phenylmethylsulfonyl fluoride (PMSF) with 1% Triton X-100). Resuspended cells were lysed by three passages through a french press cell disrupter at ~1,000 psi. Lysed cells were centrifuged for 40 min at 15,000 g, 4 ˚C, and the supernatant was discarded. The pellet (inclusion bodies) was then washed by resuspending and vortexing five times with histone wash buffer (50 mM Tris-HCl at pH 7.5, 100 mM NaCl, 1 mM EDTA). After the removal of all the supernatants, the pellets were dissolved in ion exchange (IEX) buffer A (7 M urea, 10 mM Tris pH 7.8, 1 mM EDTA, and 5 mM BME), with vortexing and sonication to assist the solubilization as much as possible. After centrifuging for 30 min at 20,000 g, 4 ˚C, the supernatant was diluted 1:1 with IEX buffer A, and then loaded onto a tandem Q-SP column (5 mL HiTrap Q HP and 5 mL HiTrap SP HP, GE healthcare). IEX buffer A containing 100 mM NaCl was used to equilibrate and wash both Q-SP column. After the removal of Q column, a gradient wash and elution of SP column with IEX buffer A containing 100 mM - 500 mM NaCl was carried out by manual mixing of IEX buffer A and IEX buffer B (7 M urea, 10 mM Tris pH 7.8, 1 mM EDTA, 1 M NaCl and 5 mM BME). All fractions containing C-H2B was combined after analyzing each fraction by Coomassie stained SDS-PAGE. The pooled C-H2B solution was concentrated by Amicon centrifugal concentrator (3.5 K MWCO, EMD Miniport), then purified by reverse-phase HPLC with a C8 semi-prep column (Varian Dynamax Microsorb 100, 250×21.4 mm). A linear gradient from 30 % CH_3CN/0.05 % TFA to 50 % CH_3CN/0.05 % TFA over 30 min at a flow rate of 10 mL/min was used for C-H2B purification to reach a final > 95 % purity by Coomassie stained SDS-PAGE. The combined solution was then lyophilized to dryness and stored at -80 ˚C until usage.

9. W4 sortase-catalyzed histone H2B semisynthesis

W4 sortase-catalyzed H2B semisynthesis followed a similar protocol as reported for F40 sortase catalyzed H3 semisynthesis[8]. The modified histone N-terminal peptide H2B 4-52 TOG was made into 5 mM stock solution, and C-H2B (aa53-125) was made into 1 mM stock solution. Both solutions were centrifuged to remove precipitate if necessary. Small scale (20 μL) W4 sortase-catalyzed reaction tests were carried out with increased amount of N-H2B peptide or W4 sortase for condition optimization (Figure S3). With the optimized condition, large scale (~5 mL) W4 sortase-catalyzed H2B semisynthesis was then carried out as below. To a sortase reaction buffer (20 mM PIPES at pH 7.5, 1 mM DTT, without the addition of 5 mM $CaCl_2$), H2B aa4-52 TOG (~1 mM) and C-H2B (~0.1 mM) were added and mixed. The ligation reaction was then initiated by the addition of W4 sortase (~500 mM) and was incubated at 37 ˚C for 12 hr. The reaction produces substantial precipitate, which contains most of the H2B product, C-H2B with some W4 sortase, and could be isolated by centrifugation. The precipitate was dissolved in 1 mL IEX buffer A (7 M urea, 10 mM Tris at pH 7.8, 1 mM EDTA and 5 mM BME) and combined with the reaction supernatant. The combined solution was purified by reverse-phase HPLC with a C8 semi-prep column (Varian Dynamax Microsorb 100, 250×21.4mm), with a linear gradient from 25 % CH_3CN/0.05 % TFA to 55 % CH_3CN/0.05 % TFA over 30 min at a flow rate of 10 mL/min. Semi-preparative RP-HPLC removed most of the W4 sortase and N-H2B, however, the fractions of FL-H2B product still contain some C-H2B (Figure S4). These product fractions were carefully pooled and lyophilized until dry. The semi-pure dry powder was dissolved in 10 % CH_3CN/ 0.05% TFA in H2O/0.05 % TFA, and repurified by reverse-phase HPLC with a C18 analytic column (Agilent, Eclipse XDB-C18, 4.6×250 mm), using a linear gradient from 30 % CH_3CN/0.05 % TFA to 50 % CH_3CN/0.05 % TFA over 30 min at a flow rate of 1 mL/min. The fractions were characterized by MALDI-TOF MS and pooled, then lyophilized to powder and stored at -80 ˚C. Purity of all semisynthesized histone H2B proteins was checked by RP-HPLC with A C18 analytic column (Agilent,

Eclipse XDB-C18, 4.6×250 mm) with a linear gradient from 5 % CH_3CN/0.05 % TFA to 95 % CH_3CN/0.05 % TFA over 30 min at a flow rate of 1 mL/min, and SDS-PAGE (Figure 2D). WT-H2B, C-H2B, H2BK11ac, H2BK12ac, H2BK11lac, and H2BK11bhb were further characterized by MALDI-TOF (Dana Farber Cancer Institute Molecular Biology Core Facilities, 4800 MALDI TOF/TOF, Applied Biosystems/MDS Sciex), while H2BK20ac and H2BK46ac were characterized by ESI-MS (LTQ Orbitrap, Thermo Q Exactive) (Figure S5).

WT-H2B: $[M + H]^+$ calculated as m/z 13494.7, observed as m/z 13493.2;
C-H2B (gH2B): $[M + H]^+$ calculated as m/z 7940.1, observed as m/z 7939.5;
H2BK11ac: $[M + H]^+$ calculated as m/z 13536.7, observed as m/z 13541.2;
H2BK12ac: $[M + H]^+$ calculated as m/z 13536.7, observed as m/z 13538.6;
H2BK20ac: $[M + H]^+$ calculated as m/z 13536.7, deconvolution as m/z 13537.8;
H2BK46ac: $[M + H]^+$ calculated as m/z 13536.7, deconvolution as m/z 13537.9;
H2BK11lac: $[M + H]^+$ calculated as m/z 13568.7, observed as m/z 13571.7;
H2BK11bhb: $[M + H]^+$ calculated as m/z 13582.7, observed as m/z 13582.8;

10. Octamer refolding and nucleosome reconstitution

In vitro octamer refolding and nucleosome assembly were carried out as previously reported.[11] Briefly, all four histone proteins (H2A, H2B, H3 and H4) were dissolved with gentle pipetting in unfolding buffer (7 M guanidine, 20 mM Tris-HCl at pH 7.5 and 10 mM DTT) for more than 30 min, but less than 3 hr, and quantified by reading A280. Octamer preparations containing H3K9ac or H3K14ac were mixed in a 1.2 : 1.2 : 1 : 1.1 ratio of H2A:H2B:H3ac:H4, making the final protein concentration was ~1 mg/mL. Octamer preparations containing acylated H2B were in a 1.1 : 1 : 0.9 : 0.9 ratio of H2A:H2Bac:H3:H4, making the final protein concentration was ~1 mg/mL. The solution was then dialyzed (10 kDa molecular weight cutoff (MWCO) cassette, Slide-a-lyzer) against high salt octamer refolding buffer (20 mM Tris at pH 7.5, 2.0 M NaCl, 1 mM EDTA and 5 mM BME) three times at 4 ˚C. Crude octamers were concentrated (10 kDa MWCO, Amicon Ultra 0.5 mL, EMD Millipore) at 4 ˚C and purified by size exclusion FPLC (AKTApurifier, GE Healthcare) using a Superdex 200 10/300 GL column (GE Healthcare) with octamer refolding buffer as the mobile phase. Preparation of 146 bp Widom 601 DNA was from the restriction digestion of a specially designed DNA plasmid from *E. coli* as previously reported methods used for nucleosome reassembly.[8] Purified octamer was mixed with Widom 601 DNA in 1:1 molar ratio in high salt (RB high) buffer (10 mM Tris at pH 7.5, 2.0 M KCl, 1 mM EDTA and 1 mM DTT) with a final concentration of 6 μM DNA. A Minipuls two channel peristaltic pump (Gilson) was used to continuously add low salt (RB low) buffer (10 mM Tris at pH 7.5, 250 mM KCl, 1 mM EDTA and 1 mM DTT) to the dialysis chamber, while simultaneously removing buffer from the chamber. The crude nucleosomes were purified by HPLC (Waters, 1525 binary pump, 2489 UV-Vis detector) with a TEKgel DEAE-5PW ion exchange column (TOSOH Bioscience, #83W-00096C) to remove free DNA. Mobile phase buffers were A TES250 (10 mM Tris 7.5, 250 mM KCl, 0.5 mM EDTA) and B TES600 (10 mM Tris 7.5, 600 mM KCl, 0.5 mM EDTA). The purification gradient was 0 % B wash for 4 min, followed by a linear gradient from 25 % to 75 % B over 30 min at 1 mL/ min flow rate. The fractions containing nucleosome products were immediately diluted with TCS Buffer (20 mM Tris at pH 7.5 and 1 mM DTT, no EDTA), then concentrated (10 kDa MWCO Amicon Ultra 4 mL, EMD Millipore) at 4 ˚C, and dialyzed three times against TCS buffer. Dialyzed nucleosomes were concentrated to 1-5 mM (Amicon Ultra 4 mL, 10 kDa MWCO, EMD Millipore), and the final nucleosome products were analyzed by native 4-20 % TBE gels (Novex™, Thermo Fisher Scientific EC62252BOX) under 120-125 Volts for 40-50 min. All the nucleosome products were over 95 % purity (free 146 bp DNA band had fluoresces 2.5-fold more brightly than the nucleosome DNA band in this setting)[12] (Figure 2E).

11. Expression and purification of HDAC complexes

The expression and purification of most HDAC complexes were performed as described previously.[8] For each complex, two to four plasmids corresponding to the different components of the HDAC complex were co-transfected into HEK293F cells. After the cell lysis by sonication, each complex was purified by FLAG

tag immunoaffinity chromatography and then cleaved from the resin by TEV protease. The mixture was purified further using gel filtration on either a Superdex 200 Increase 10/ 300 GL column (MiDAC, RERE, MIER1) or a Superose 6 10/300 GL column (CoREST, NuRD/HMMR, Sin3A) for further purification. The purified complexes were finally concentrated to 1–5 mM and analyzed by SDS-PAGE stained with Coomassie to confirm purity (>80 %). The complexes were stored at -80˚C until further use after the addition of final glycerol concentration to 10 % (Figure S6A).
Flag tagged CoREST1 (aa83–482), HDAC1 (FL aa1–482), and LSD1 (aa1–852) for the CoREST complex;
Flag tagged MTA1 (FL aa1–715), HDAC1 (FL aa1–482), RBBP4 (FL aa1–425), and MBD2 (aa145-411) for the HMMR complex;
Flag tagged Sin3A (aa461-1273), HDAC1 (FL aa1–482), RBBP4 (FL aa1–425), Sap30L (FL aa1-183) for the Sin3A complex;
Flag tagged MIDEAS (aa628-887), HDAC1 (FL aa1–482), DNTTIP1 (FL aa1–329) for the MiDAC complex;
Flag tagged MIER1 (FL aa1-512), HDAC1 (FL aa1-482) for the MIER1 complex;
Flag tagged RERE (aa1-565), HDAC1 (FL aa1-482) for the RERE complex.

12. Analysis of HDAC deacylation of acylated H2B & H3 nucleosomes and histones

For all the deacetylation assays by HDAC complexes, each of the semisynthetic free histone proteins (H3K9ac, H2BK11ac, H2BK12ac, H2BK20ac, H2B46ac, H2BK11lac, and H2BK11bhb) (final 1.0 μM) or the corresponding nucleosomes (H3K9ac, H3K14ac, H2BK11ac, H2BK12ac, H2BK20ac, H2B46ac, H2BK11lac, and H2BK11bhb) (final 100 nM) were mixed with a HDAC reaction buffer (50 mM HEPES at pH 7.5, 100 mM KCl, 0.2 mg/mL BSA, and 100 μM inositol hexaphosphate (IP_6)[13]). The reaction solution was kept on ice until the addition of different HDAC1 complexes at different final enzyme concentrations, and then incubated at 37 ˚C. The complexes include CoREST, HMMR, Sin3A, MiDAC, MIER, and RERE, as well as free HDAC1 enzyme (BPS Bioscience). At different time points, multiple reaction samples were taken. In a typical sample, 8 μL aliquots of the reaction were first taken and quenched with gel loading buffer (4 μL 40 mM EDTA, 4 μL 4 x Laemmli sample buffer). Each sample would then be boiled for 3-5 min at 95 ˚C and then resolved on a 4-20 % gradient SDS-PAGE gel (TGX™, Bio-Rad, 4561096) at 180 Volts for ~20 min. Gels were transferred to nitrocellulose membrane (Transfer Stack, Invitrogen, IB301031) for western blot analysis (WB) by iBlot (Invitrogen) (P3, 5.0 min). Site-specific antibodies for acetylated H3 and acylated H2B, such as for anti-H3K9ac, anti-H3K14ac, anti-H2BK11ac (Invitrogen, #PA5-112479), anti-H2BK12ac (ActiveMotif, #39669), anti-H2BK20ac (Revmab Biosciences #31-1113-00), anti-H2BK46ac (ActiveMotif, #39571), anti-H2BK11lac (provided by the Yingming Zhao lab from PTM Bio, Inc. (Chicago, IL)), and anti-H2BK11bhb (provided by the Yingming Zhao lab from PTM Bio, Inc. (Chicago, IL)) were used to blot the membrane. At the same time, anti-H3 or anti-H2B (Abcam, #ab134211, or Abcam, #ab52484) was used to visualize total H3 or H2B, respectively. The affinities and specificities of the primary H2B antibodies were tested and validated (Figure S7), while the specificities of the primary antibodies anti-H3K9ac and anti-H3K14ac have been confirmed previously[8]. After secondary antibody Anti-Rabbit IgG, HRP linked antibody (Cell signaling #7074S) blotting, the membranes were treated with the ECL substrate (Bio-Rad), and visualized by the G:BOX mini gel imager (Syngene). The bands were then quantified by ImageJ, and all intensity values were fit to a single-phase exponential decay curve with constrain Y0=1, Plateau=0 (GraphPad Prism Nine). Each plotted point represents at least 2 replicates (Figure S10-16). The kinetic parameter V/[E] was calculated using GraphPad Prism Nine. Heatmap visualization of all calculated V/[E] values were plotted with GraphPad Prism Nine (Figure 4AB). Normalized V/[E] were calculated for histone assays (V/[E] divided by a factor of 5) given that each nucleosome contains two H3 molecules and the final histone protein substrate (1.0 μM) is ten times more than nucleosome substrate (100 nM) (Table S2-3). The remaining nucleosome samples after HDAC1 complex assay were analyzed by native 4-20 % TBE gels in comparison with the samples before assay to confirm the stability of nucleosome throughout the assay (Figure S8A).

13. SAHA inhibition assay of CoREST on H2BK11lac and H2BK11bhb proteins

To verify that the removal of H2BK11lac and H2BK11bhb was enzymatic, we carried out the deacetylation assays on H2B proteins using the CoREST complex with or without the HDAC inhibitor SAHA (Vorinostat). SAHA (Sigma, SML0061-5MG) was dissolved in 0.25 % DMSO in water to 1 mM. Pure water containing 0.25 % DMSO was used as a vehicle control. 100 μM SAHA was added to the HDAC reaction buffer containing 1.2 μM of CoREST complex and was incubated for 15 min at room temperature, before incubating for another 2 min on ice. The deacetylation reaction was initiated with the addition of H2BK11lac or H2BK11bhb protein to a final concentration of 1.0 μM. The final reaction mixture contained 10 μM SAHA, 1.0 μM H2B protein, 120 nM CoREST, and 0.0025 % DMSO, while the vehicle control contains 1.0 μM H2B protein, 120 nM CoREST, and 0.0025 % DMSO. After thorough mixing, the reactions were incubated at 37 ˚C, before processing as described above (Figure S18).

14. Comparison of NuRD and HMMR deacetylation kinetics on H3K9ac nucleosome

We previously characterized deacetylation of H3 by the NuRD complex (HDAC1, MTA1, and RBBP4/7). In order to compare the data collected here with the expanded HMMR complex (HDAC1, MTA1, RBBP4/7, and MBD2), we carried out parallel assays on H3K9ac nucleosomes using HMMR and NuRD.[8] Assays were conducted as described in the general HDAC assay protocol, and all data was analyzed as described above (Figure S13A).

15. Fluor de Lys assay on commercial HDAC1

HDAC1 (1-482(end)-His-FLAG) in complex with Hsp70 (BPS Bioscience, #50051) (Figure S6A) was purchased with Fluorogenic HDAC Assay Kit (BPS Bioscience, #50031), and its activity was confirmed with the Fluor de Lys assay. HDAC1 was first mixed with HDAC reaction buffer to a 300 nM stock solution, while the HDAC peptide substrate 3 (BPS Bioscience) was diluted with sirtuin reaction buffer to a 500 nM stock solution. To a black 96-well plate (MicroWell™, Thermo, #267342), a final concentration of 50 nM peptide substrate was mixed with a concentration gradient of HDAC1 (0, 1 nM, 2 nM, 4 nM, 8 nM, 16 nM) within separate wells, while a well only containing HDAC reaction buffer was used as the negative control. After being covered with aluminum foil, the plate was incubated for 30 min at 37 ˚C, followed by the addition of HDAC Assay Developer (2×, BPS Bioscience, #50030). After well-mixed, the plate was incubated for 15 min at room temperature. The samples were read by microtiter-plate reading fluorimeter (BioTek Cytation 5) with 365 nm excitation and 450 nm emission. The data were exported and plotted in linear fitting after subtracting the negative control (GraphPad Prism Nine), with 2 replicates for each data point (Figure S19A).

16. Cloning, expression, and purification of sirtuins

The expression and purification of human sirtuins[14] generally followed the reported protocol[15,16]. The purified sirtuins were concentrated to 3–20 mM and analyzed by SDS-PAGE stained with Coomassie to confirm purity (> 90 %). The aliquots were stored at -80˚C until further use after the addition of final glycerol concentration to 10 % (Figure S6AB).
Removed N-terminal His-Sumo tag Sirt2: (aa56-356);
N-terminal non-cleavable 6xHis Sirt3: (aa101-399);
Sirt5-Flag tagged: (FL, aa1-310).

17. Fluor de lys assay on commercial Sirt1

Sirt1 (GST-aa193-741) was purchased with the Fluorogenic Sirt1 Assay Kit (BPS Bioscience, #50081), and its activity was confirmed with the Fluor de Lys assay. Sirt1 was first mixed with a sirtuin reaction buffer (50 mM HEPES at pH 7.5, 1 mM DTT, 0.2 mg/mL BSA, and 1 mM NAD^+)[14] to give a 900 nM stock solution, while the sirtuin peptide substrate (BPS Bioscience) was diluted with sirtuin reaction buffer to give a 500 nM stock solution. To a black 96-well plate (MicroWell™, Thermo, #267342), a final concentration of 50 nM peptide substrate was mixed with a concentration gradient of Sirt1 (0, 10 nM, 30 nM, 90 nM) in separate wells, while a well only containing sirtuin reaction buffer was used as the negative

control. The plate was covered with aluminum foil, and incubated for 30 min at 37 ˚C, followed by the addition of sirtuin developer (2×, BPS Bioscience). After thorough mixing, the plate was incubated for 15 min at room temperature. The samples were read by microtiter-plate reading fluorimeter (BioTek Cytation 5) with 365 nm excitation and 450 nm emission. The data were exported and plotted in linear fitting after subtracting the negative control (GraphPad Prism Nine), with 2 replicates for each data point (Figure S19B).

18. Analysis of sirtuin deacylation of acylated H2B & H3 nucleosomes and histones

The general setup and processing of all the deacetylation assays by sirtuins were similar to the HDAC complex assays described above. The nucleosome or histone substrates was mixed with the sirtuin reaction buffer (50 mM HEPES at pH 7.5, 1 mM DTT, 0.2 mg/mL BSA, and NAD^+ 1 mM)[14], and the deacylation reactions were initiated with the addition of Sirt1, Sirt2, Sirt3, and Sirt5 to different final concentrations (Figure S20-23). A decrease in total H2B was observed in each assay with free histone H2B protein, thus all intensity values were normalized according to the corresponding anti-H2B intensity value. Normalized data was fit to a single-phase exponential decay curve software with constrain Y0=1, Plateau=0 (GraphPad Prism Nine) (Table S2-3). The remaining nucleosome samples after sirtuin assay were analyzed by native 4-20 % TBE gels in comparison with the samples before assay to confirm the stability of nucleosome throughout the assay (Figure S8B).

19. WT-H2B and WT-H3 assay with different concentration of sirtuins

We hypothesize that the protein loss during assays with the sirtuins was due to the aggregation of histone protein, which was in a thermodynamically unstable state. To rule out that the observed decrease in total H2B protein during sirtuin assays was due to the addition of sirtuin, or as an antibody issue, we carried out an aggregation test with WT-H2B and WT-H3 with a concentration gradient of sirtuin. In this assay, Sirt2 was specifically used due to its robust activity on both H2Bac nucleosomes and free histone H2Bac proteins. WT-H2B (final 1.0 µM) or WT-H3 (final 1.0 µM) was mixed with the sirtuin reaction buffer, followed by the addition of a concentration gradient of Sirt2 (0, 30 nM, 120 nM). The reaction solutions were incubated at 37 ˚C, and aliquots were removed at multiple subsequent time points. Western blotting was as described above. Samples were further characterized by SDS-PAGE gel (4-20 % TGX™, Bio-Rad, 4561096) stained by Colloidal Blue Staining (Invitrogen, #LC6025) (Figure S9).

20. Enzyme concentration-dependent assay for HDAC1 complexes and sirtuins

For the MIER complex, an enzyme concentration-dependent assay was carried out for both H3K9ac nucleosomes and free protein. For nucleosome deacetylation assays, two different final concentrations of the MIER complex (120 nM, 30 nM) were mixed with H3K9ac nucleosomes (Figure S17A) and H2BK11ac nucleosomes (data not shown, due to undetectable activity at 30 nM enzyme). The velocity showed a non-linear relationship with enzyme concentration (leveling off at high MIER complex concentration), thus the V/[E] from the 30 nM group was decided to be more accurate. For histone deacetylation assays, seven different final concentrations of MIER (120 nM, 60 nM, 30 nM, 10 nM, 5 nM, 1 nM, 0.2 nM) were mixed with free histone H3K9ac protein (Figure S17B). The velocity also showed a non-linear relationship with the enzyme concentration (leveling off at high MIER complex concentration), thus the V/[E] from the 0.2 nM group was decided to be most accurate. Several other enzyme concentration-dependent assays were also carried out: MiDAC (120 nM, 30 nM) with H2BK11lac nucleosome (Figure S12F); MiDAC (120 nM, 30 nM) with H2BK11bhb nucleosome (Figure S12G); RERE (120 nM, 30 nM) with H3K9ac nucleosome (Figure S16A); Sirt2 (60 nM, 30 nM) with H3K9ac nucleosome (Figure S21A); Sirt1 (20 nM, 5 nM) on free histone H3K9ac protein (Figure S20A). In each of these cases, the velocity measurements generally showed a linear relationship with enzyme concentration.

21. Complex-specific rate-weighted ranking of local sequences

The rates of individual HDAC complexes were normalized to the lowest measured rate, which was set to a value of 1. All normalized values were then rounded to the nearest integer. Each deacetylation site was defined by the five amino acids preceding, and 5 amino acids following the target lysine. These acetylation

sites were FASTA formatted and compiled into a list. The number of times that a site appears in the list was determined by the normalized integer value of the rate a complex exhibited toward that site. The WebLogo tool[17] (http://weblogo.berkeley.edu/logo.cgi) for consensus sequence visualization was used to convert FASTA lists into plots illustrating rate-weighted amino acid frequency surrounding an acetylation site (Figure S24, Figure S25).

Supplemental References:

(1) Chen, I.; Dorr, B. M.; Liu, D. R. A general strategy for the evolution of bond-forming enzymes using yeast display. *Proc Natl Acad Sci U S A*. **2011**, *108* (28), 11399–11404.

(2) Piotukh, K.; Geltinger, B.; Heinrich, N.; *et al.* Directed Evolution of Sortase A Mutants with Altered Substrate Selectivity Profiles. *J Am Chem Soc*. **2011**, *133* (44), 17536–17539.

(3) Hirakawa, H.; Ishikawa, S.; Nagamune, T. Design of Ca 2+-independent Staphylococcus aureus sortase A mutants. *Biotechnol Bioeng*. **2012**, *109* (12), 2955–2961.

(4) Jeong, H. J.; Abhiraman, G. C.; Story, C. M.; Ingram, J. R.; Dougan, S. K. Generation of Ca2+-independent sortase A mutants with enhanced activity for protein and cell surface labeling. *PLoS One*. **2017**, *12* (12), 1–15.

(5) Williamson, D. J.; Fascione, M. A.; Webb, M. E.; Turnbull, W. B. Efficient N-terminal labeling of proteins by use of sortase. *Angew Chemie Int Ed*. **2012**, *51* (37), 9377–9380.

(6) Wu, M.; Hayward, D.; Kalin, J. H.; Song, Y.; Schwabe, J. W.; Cole, P. A. Lysine-14 acetylation of histone H3 in chromatin confers resistance to the deacetylase and demethylase activities of an epigenetic silencing complex. *Elife*. **2018**, *7*, 1–19.

(7) Luger, K.; Rechsteiner, T. J.; Richmond, T. J. Preparation of Nucleosome Core Particle from Recombinant Histones. *Methods Enzymol*. **1999**, *304* (1997), 3–19.

(8) Wang, Z. A.; Millard, C. J.; Lin, C.-L.; *et al.* Diverse nucleosome site-selectivity among histone deacetylase complexes. *Elife*. **2020**, *9*, e57663.

(9) Jani, K. S.; Jain, S. U.; Ge, E. J.; *et al.* Histone H3 tail binds a unique sensing pocket in EZH2 to activate the PRC2 methyltransferase. *Proc Natl Acad Sci U S A*. **2019**, *116* (17), 8295–8300.

(10) Gatchalian, J.; Wang, X.; Ikebe, J.; *et al.* Accessibility of the histone H3 tail in the nucleosome for binding of paired readers. *Nat Commun*. **2017**, *8* (1), .

(11) Dyer, P. N.; Raji, E. S.; White, C. L.; *et al.* Reconstitution of Nucleosome Core Particles from Recombinant Histones and DNA. *Methods Enzymol*. **2004**, *375*, 23–44.

(12) Shahian, T.; Narlikar, G. J. Chapter 20: Analysis of Changes in Nucleosome Conformation Using Fluorescence Resonance Energy Transfer. *Chromatin Remodel. Methods Protoc.* Vol. 833. **2012**:337–349.

(13) Watson, P. J.; Millard, C. J.; Riley, A. M.; *et al.* Insights into the activation mechanism of class i HDAC complexes by inositol phosphates. *Nat Commun*. **2016**, *7* (11262), 1–13.

(14) Spiegelman, N. A.; Price, I. R.; Jing, H.; *et al.* Direct Comparison of SIRT2 Inhibitors: Potency, Specificity, Activity-Dependent Inhibition, and On-Target Anticancer Activities. *ChemMedChem*. **2018**, *13* (18), 1890–1894.

(15) Jing, H.; Hu, J.; He, B.; *et al.* A SIRT2-Selective Inhibitor Promotes c-Myc Oncoprotein Degradation and Exhibits Broad Anticancer Activity. *Cancer Cell*. **2016**, *29* (3), 297–310.

(16) Du, J.; Zhou, Y.; Su, X.; *et al.* Sirt5 is a NAD-dependent protein lysine demalonylase and desuccinylase. *Science*. **2011**, *334* (6057), 806–809.

(17) Crooks, G. E.; Hon, G.; Chandonia, J. M.; Brenner, S. E. WebLogo: A sequence logo generator. *Genome Res*. **2004**, *14* (6), 1188–1190.

(18) Suree, N.; Liew, C. K.; Villareal, V. A.; *et al.* The structure of the Staphylococcus aureus sortase-substrate complex reveals how the universally conserved LPXTG sorting signal is recognized. *J Biol Chem*. **2009**, *284* (36), 24465–24477.

(19) DeLano, W. L. The PyMOL Molecular Graphics System, Version 2.3. *Schrödinger LLC*. **2020**.

(20) Lee, G. R.; Won, J.; Heo, L.; Seok, C. GalaxyRefine2: Simultaneous refinement of inaccurate local regions and overall protein structure. *Nucleic Acids Res*. **2019**, *47* (W1), W451–W455.

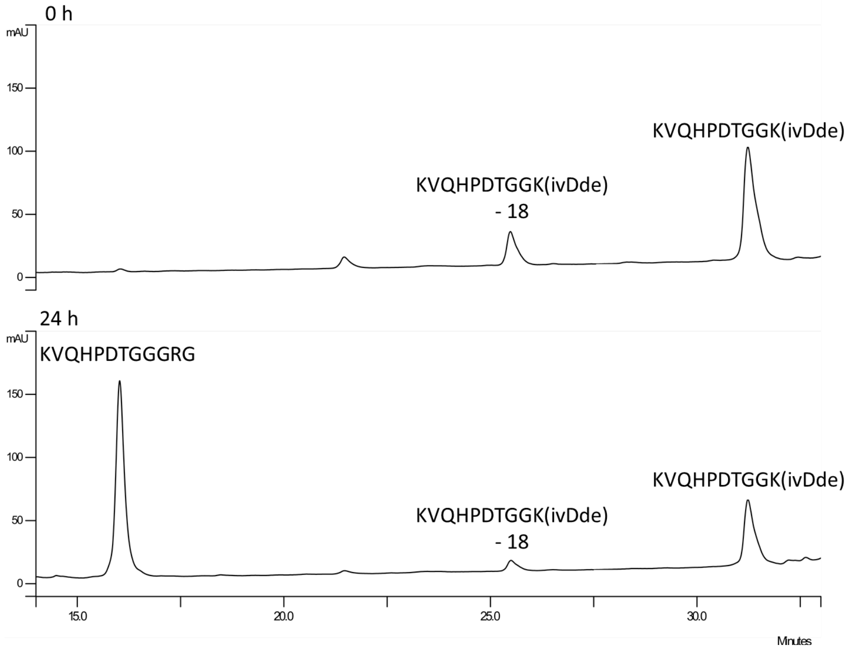

22. Figure S1. Representative RP-HPLC chromatogram of model peptide conversion.
Chromatogram from 0 hr reaction time (top), and 24 hr reaction time (bottom). Peaks corresponding to the transpeptidation product KQVHPDTGGGRG, and starting materials KQVHPDTGGK(ivDde) and KQVHPDTGGK(ivDde) - 18 Da are indicated.

23. Table S1. Activity of sortase mutants on a model peptide substrate.
Decrease in the area under the curve (214 nm) of the substrate peptide, KQVHPDTGGK(ivDde), over 24 hr reaction time (n ≥3, ± standard deviation).

Sortase mutant	Decrease in substrate
F40 (n = 3)	3.9% ± 3.4%
W1 (n = 3)	0%
W2 (n = 3)	5.2% ± 2.8%
W3 (n = 4)	4.9% ± 2.1%
W4 (n = 4)	41% ± 11%

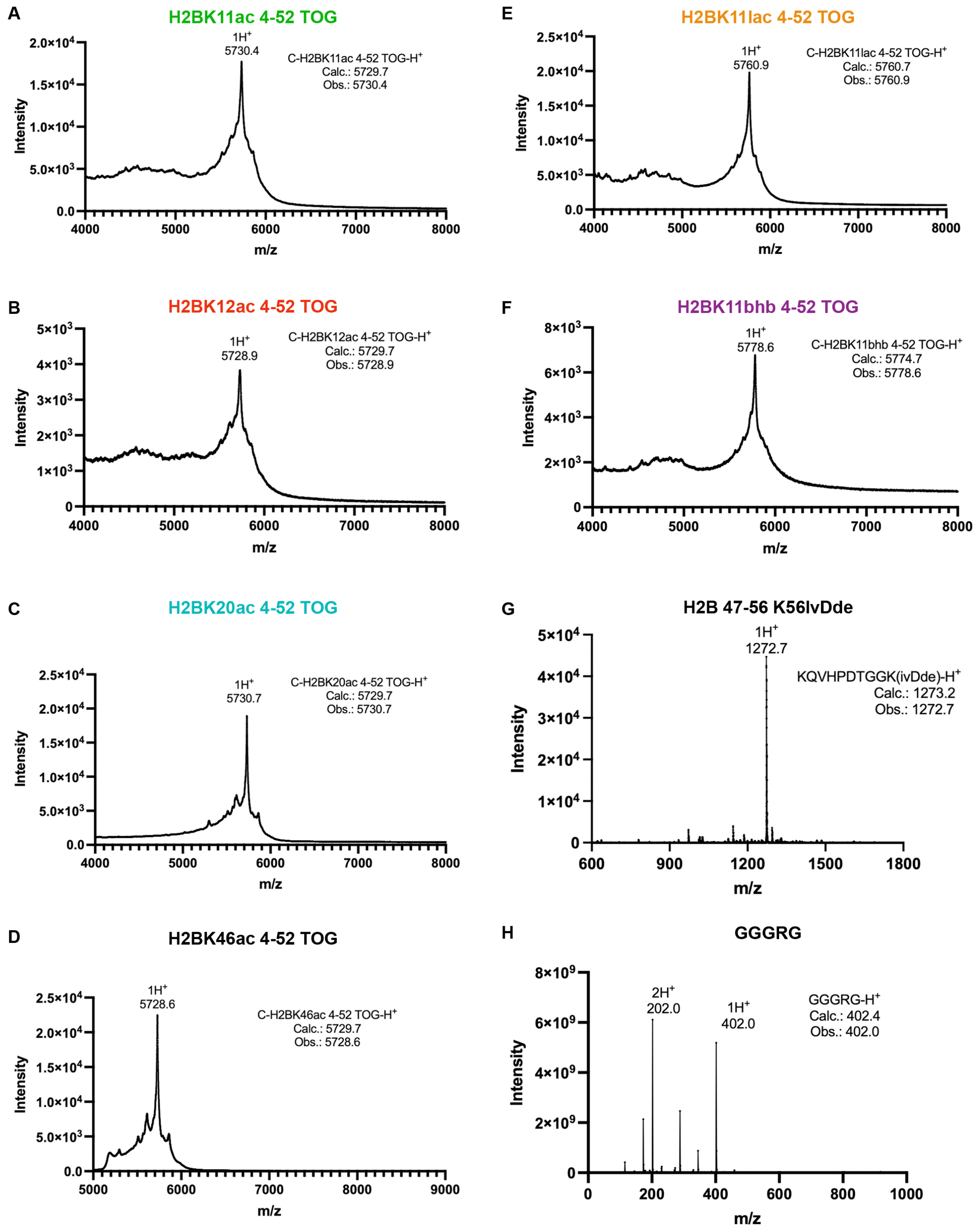

24. Figure S2. All MS for N-H2B peptides and model peptides.

MALDI-TOF for (A) H2BK11ac aa4-52 TOG peptide; (B) H2BK12ac aa4-52 TOG peptide; (C) H2BK20ac aa4-52 TOG peptide; (D) H2BK46ac aa4-52 TOG peptide; (E) H2BK11lac aa4-52 TOG peptide; (F) H2BK11bhb aa4-52 TOG peptide; (G) H2B 47-56 K56ivDde peptide; (H) ESI-MS for GGGRG peptide, also confirmed by H^1NMR (data not shown).

No.	N/A	N/A	N/A	N/A	N/A	0	1	2	3	4
Buffer		-	-	-	-	+	+	+	+	+
C-H2B		+	-	-	-	+	+	+	+	+
N-H2B		-	+	-	-	-	+	++	+	++
FL-WT-H2B		-	-	-	+	N/A	N/A	N/A	N/A	N/A
W4 sortase		-	-	+	-	++	+	+	++	++
Time/hr		N/A	N/A	N/A	N/A	12	12	12	12	12

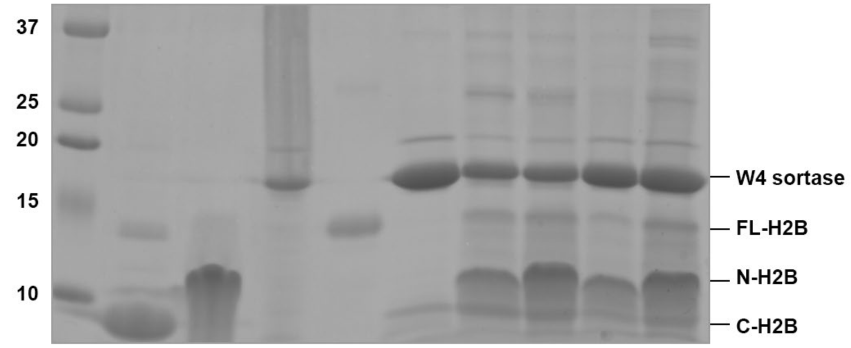

25. Figure S3. Condition optimization of W4 sortase-catalyzed histone H2B semisynthesis

Small scale (20 µl) W4 sortase-catalyzed reaction tests were carried out with 4 different conditions in a sortase reaction buffer at 37 ˚C for 12 hr, and then analyzed by SDS-PAGE (H2BK12ac as example). W4 sortase, Full-length histone H2B product (FL-H2B), substrates N-H2B aa4-52 TOG, and C-H2B (aa53-135) are indicated, while W4 sortase, FL Wild-Type histone H2B product (FL-WT-H2B), substrates N-H2B aa4-52 TOG, and C-H2B (aa53-135) are used as controls. Condition 4 was chosen to setup large scale (~5 mL) W4 sortase-catalyzed H2B semisynthesis.

Condition 0: W4 sortase (~500 µM), N-H2B aa4-52 TOG (0 mM), and C-H2B (~0.1 mM);
Condition 1: W4 sortase (~160 µM), N-H2B aa4-52 TOG (~0.3 mM), and C-H2B (~0.1 mM);
Condition 2: W4 sortase (~160 µM), N-H2B aa4-52 TOG (~1 mM), and C-H2B (~0.1 mM);
Condition 3: W4 sortase (~500 µM), N-H2B aa4-52 TOG (~0.3 mM), and C-H2B (~0.1 mM);
Condition 4: W4 sortase (~500 µM), N-H2B aa4-52 TOG (~1 mM), and C-H2B (~0.1 mM).

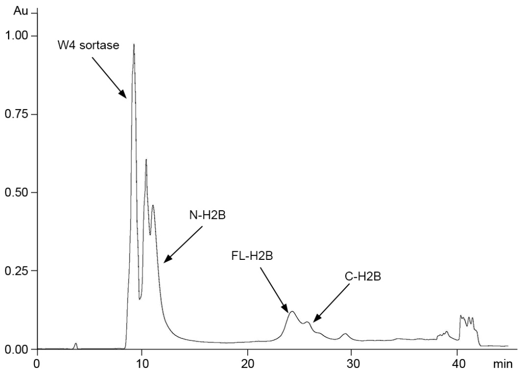

26. Figure S4. Representative semi-prep HPLC chromatography for W4 sortase-catalyzed H2B synthesis

The reaction mixture (H2BK12ac as example) was purified by reverse-phase HPLC with a C8 semi-prep column with a linear gradient from 25 % CH_3CN/0.05 % TFA to 55 % CH_3CN/0.05 % TFA over 30 min at a flow rate of 10 mL/min. Semi-preparative RP-HPLC removed most of the W4 sortase and N-H2B, however, the fractions of full-length (FL) H2B product still contained some C-H2B. Overall FL-H2B synthesis provided ~40 % yield (based on C-H2B substrate concentration).

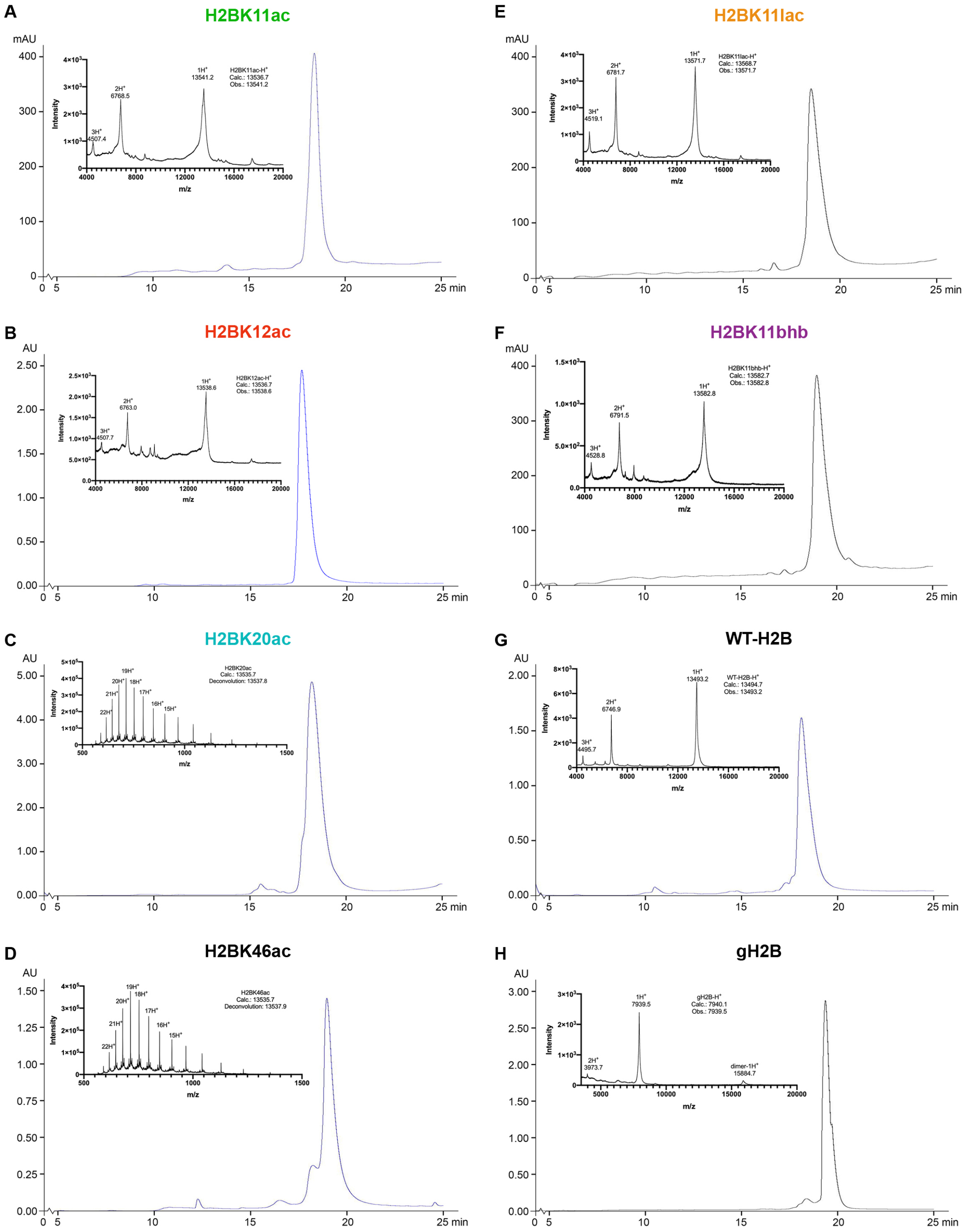

27. Figure S5. All analytic HPLC chromatography and MALDI-TOF for full-length H2B

(A) H2BK11ac aa4-125 histone protein; (B) H2BK12ac aa4-125 histone protein; (E) H2BK11lac aa4-125 histone protein; (F) H2BK11bhb aa4-125 histone protein; (G) WT-H2B aa4-125 histone protein; (H) C-H2B (gH2B) aa53-125 protein; Analytic HPLC chromatography and ESI-MS for (C) H2BK20ac aa4-125 histone protein; (D) H2BK46ac aa4-125 histone protein.

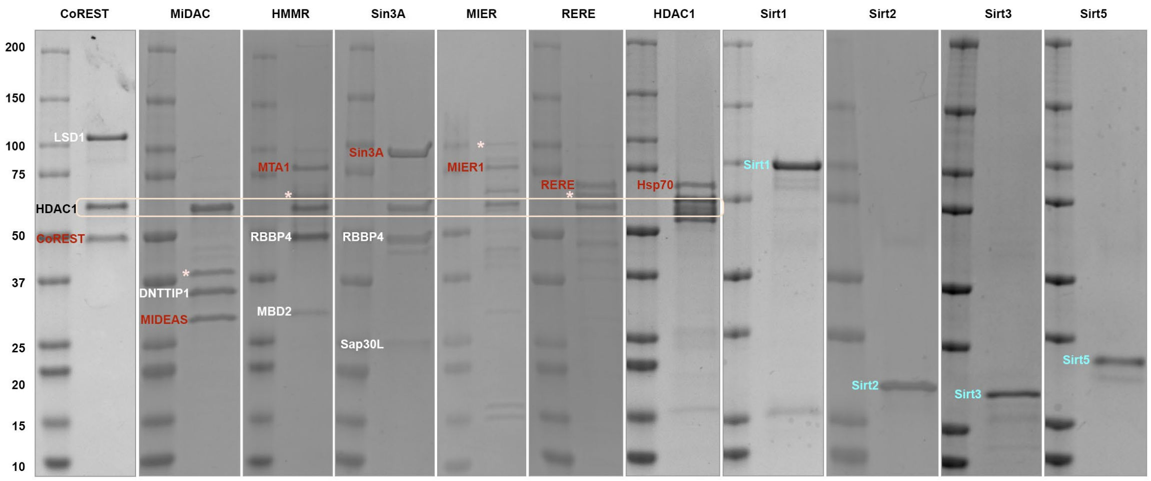

B

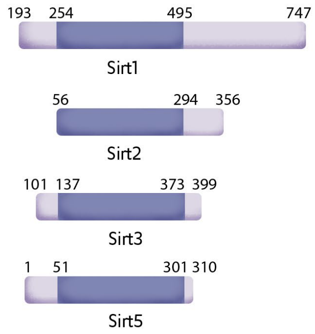

28. Figure S6. SDS-PAGE for All HDAC1 complexes and sirtuins

(A) HDAC1 in the different complexes is shown within the pink rectangle. The scaffold proteins are labeled in red, and the third components of each complex are labeled in white. The major impurity bands of each HDAC complexes are labeled as *. Each sirtuin is labeled in cyan. (B) Domain architectures for Sirt1, 2, 3, and 5.

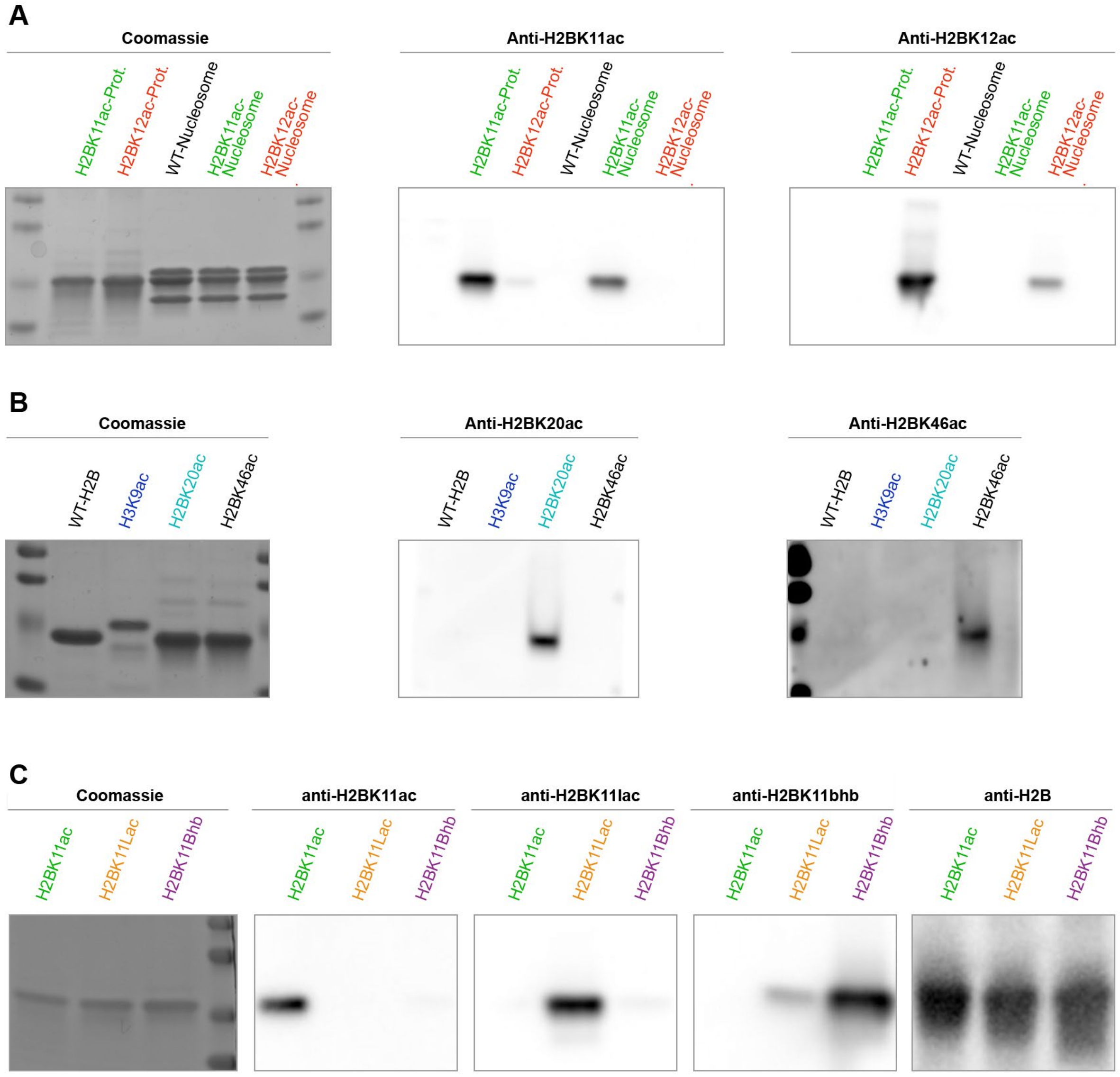

29. Figure S7. Antibody affinity and specificity tests

(A) SDS-PAGE for free histone H2BK11ac protein, free histone H2BK12ac protein, free histone WT-H2B protein, H2BK11ac nucleosome, H2BK12ac nucleosome, by Coomassie staining, or transferred to membrane followed by blotting with anti-H2BK11ac, and with anti-H2BK12ac; (B) SDS-PAGE for free histone WT-H2B protein, free histone H3K9ac protein, free histone H2BK20ac protein, free histone H2BK46ac protein, by Coomassie staining, or transferred to membrane followed by blotting with anti-H2BK20ac, and with anti-H2BK46ac; (C) SDS-PAGE for free histone H2BK11ac protein, free histone H2BK11lac protein, free histone H2BK11bhb protein, by Coomassie staining, or transferred to membrane followed by blotting with anti-H2BK11ac, anti-H2BK11lac, anti-H2BK11bhb, and anti-H2B.

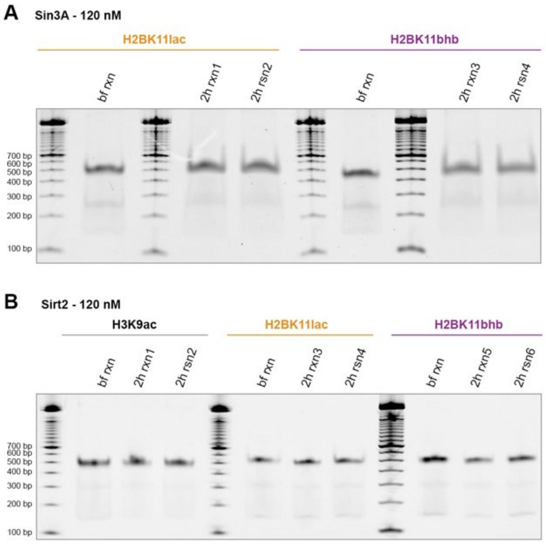

30. Figure S8 Representative TBE gel for nucleosomes before and after treatment with an HDAC complex (Sin3A) or sirtuin (Sirt2)

(A) H2BK11lac and H2BK11bhb nucleosomes before and after 120 nM Sin3A assay; (B) H3K9ac, H2BK11lac and H2BK11bhb nucleosomes before and after 120 nM Sirt2 assay.

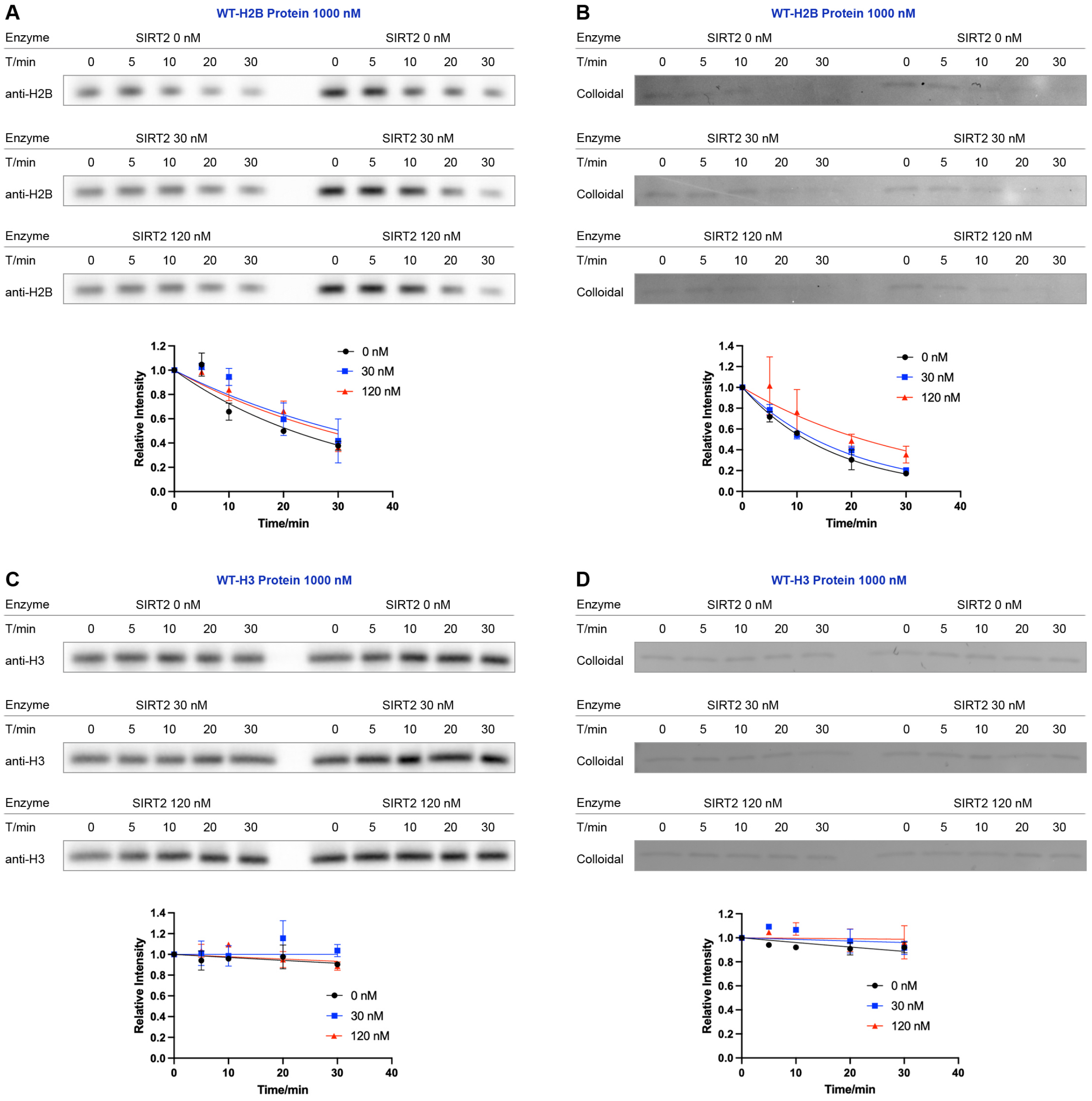

31. Fig. S9. WT-H2B and WT-H3 protein aggregation test in sirtuin reaction buffer

(A) WB and curve-fitting of WT-H2B (final 1.0 μM) mixed with a concentration gradient of Sirt2 (0, 30 nM, 120 nM) in the sirtuin reaction buffer; (B) SDS-PAGE with colloidal staining and curve-fitting of WT-H2B (final 1.0 μM) mixed with a concentration gradient of Sirt2 (0, 30 nM, 120 nM) in the sirtuin reaction buffer; (C) WB and curve-fitting of WT-H3 (final 1.0 μM) mixed with a concentration gradient of Sirt2 (0, 30 nM, 120 nM) in the sirtuin reaction buffer; (D) SDS-PAGE with colloidal staining and curve-fitting of WT-H3 (final 1.0 μM) mixed with a concentration gradient of Sirt2 (0, 30 nM, 120 nM) in the sirtuin reaction buffer.

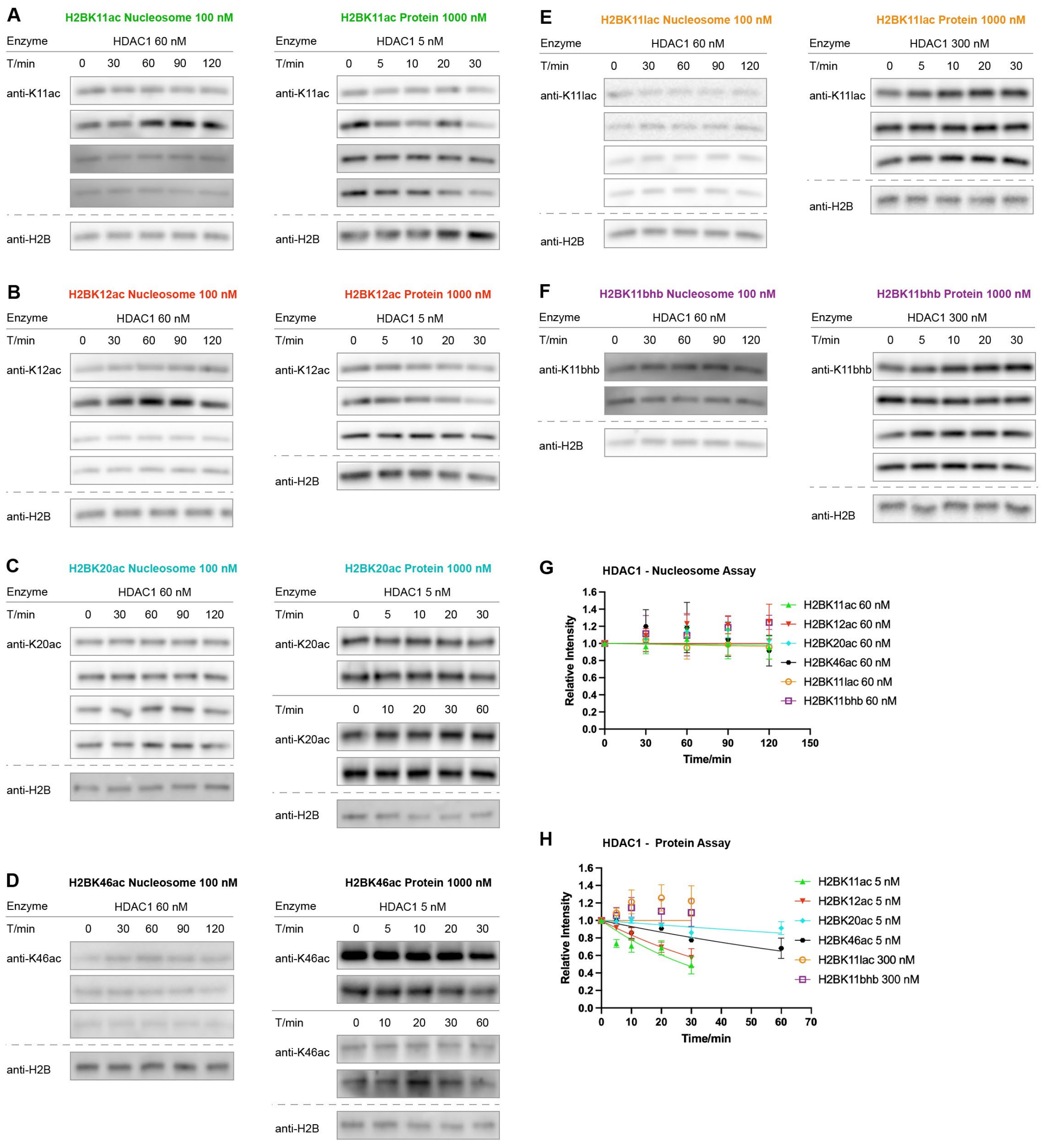

32. Figure S10. WB and curve fitting for deacylation by free HDAC1

(A) H2BK11ac (n = 4 for nucleosome assay; n = 4 for free histone protein assay); (B) H2BK12ac (n = 4 for nucleosome assay; n = 3 for free histone protein assay), (C) H2BK20ac (n = 4 for nucleosome assay; n = 4 for free histone protein assay), (D) H2BK46ac (n = 3 for nucleosome assay; n = 3 for free histone protein assay). (E) H2BK11lac (n = 4 for nucleosome assay; n = 3 for free histone protein assay); (F) H2BK11bhb (n = 2 for nucleosome assay; n = 4 for free histone protein assay); (G) Curve fitting for nucleosome kinetics with 60 nM HDAC1 labeled in the graph; (H) Curve fitting for histone kinetics with 5, or 300 nM HDAC1 labeled in the graph.

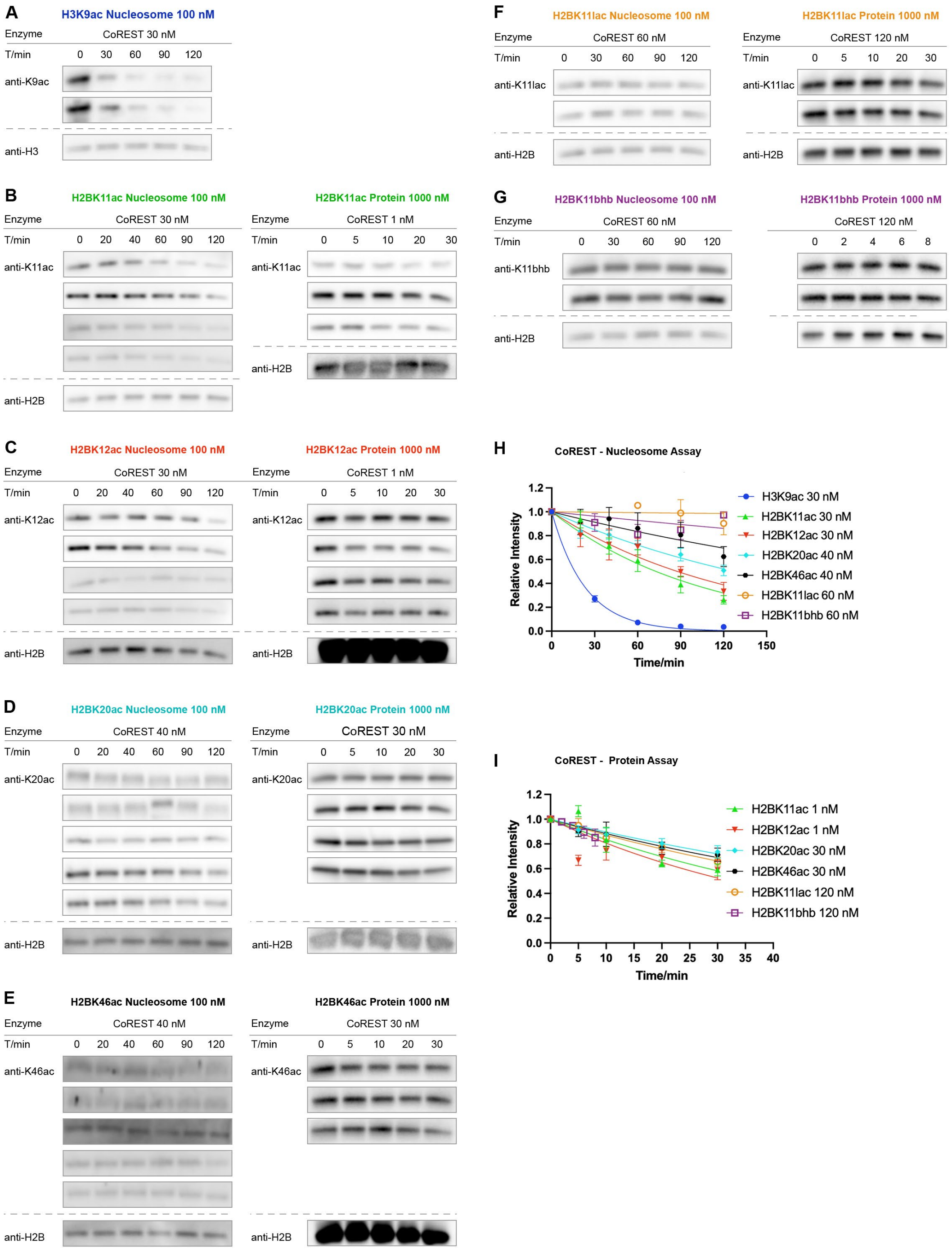

33. Figure S11. WB and curve fitting for deacylation by the CoREST complex

(A) H3K9ac (n = 2 for nucleosome assay); (B) H2BK11ac (n = 4 for nucleosome assay; n = 3 for free histone protein assay); (C) H2BK12ac (n = 4 for nucleosome assay; n = 4 for free histone protein assay), (D) H2BK20ac (n = 5 for nucleosome assay; n = 4 for free histone protein assay), (E) H2BK46ac (n = 5 for nucleosome assay; n = 3 for free histone protein assay). (F) H2BK11lac (n = 2 for nucleosome assay; n = 2 for free histone protein assay); (G) H2BK11bhb (n = 2 for nucleosome assay; n = 2 for free histone protein assay); (H) Curve fitting for nucleosome kinetics with 30, 40, or 60 nM CoREST labeled in the graph; (I) Curve fitting for histone kinetics with 1, 30, or 120 nM CoREST labeled in the graph.

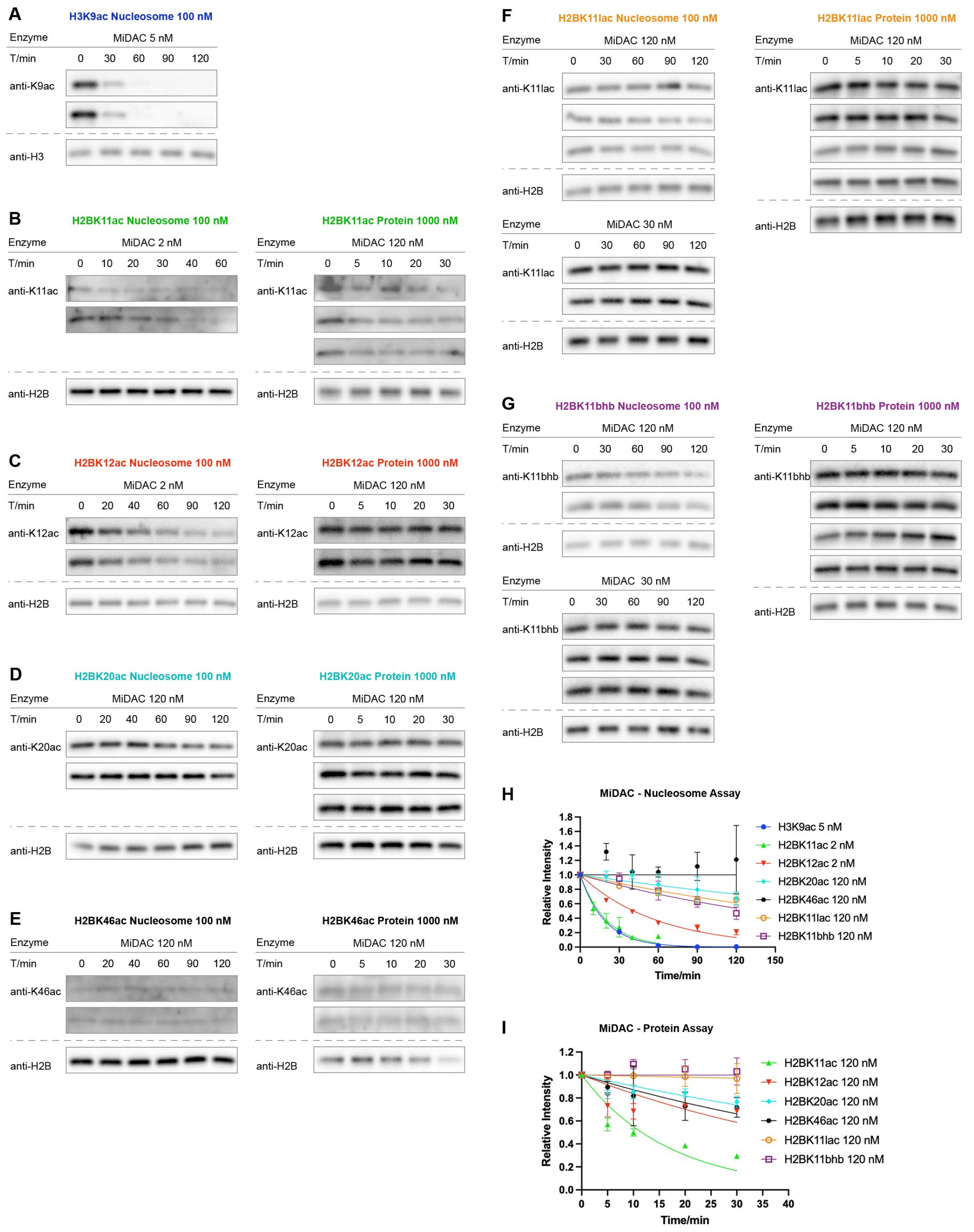

34. Figure S12. WB and curve fitting for deacylation by the MiDAC complex

(A) H3K9ac (n = 2 for nucleosome assay); (B) H2BK11ac (n = 2 for nucleosome assay; n = 3 for free histone protein assay); (C) H2BK12ac (n = 2 for nucleosome assay; n = 2 for free histone protein assay), (D) H2BK20ac (n = 2 for nucleosome assay; n = 3 for free histone protein assay), (E) H2BK46ac (n = 2 for nucleosome assay; n = 2 for free histone protein assay). (F) H2BK11lac (n = 2 for nucleosome assay with 30 nM MiDAC & n = 3 with 120 nM MiDAC; n = 4 for free histone protein assay); (G) H2BK11bhb (n = 3 for nucleosome assay with 30 nM MiDAC & n = 2 with 120 nM MiDAC; n = 4 for free histone protein assay); (H) Curve fitting for nucleosome kinetics with 2, 5, or 120 nM MiDAC labeled in the graph; (I) Curve fitting for histone kinetics with 120 nM MiDAC labeled in the graph.

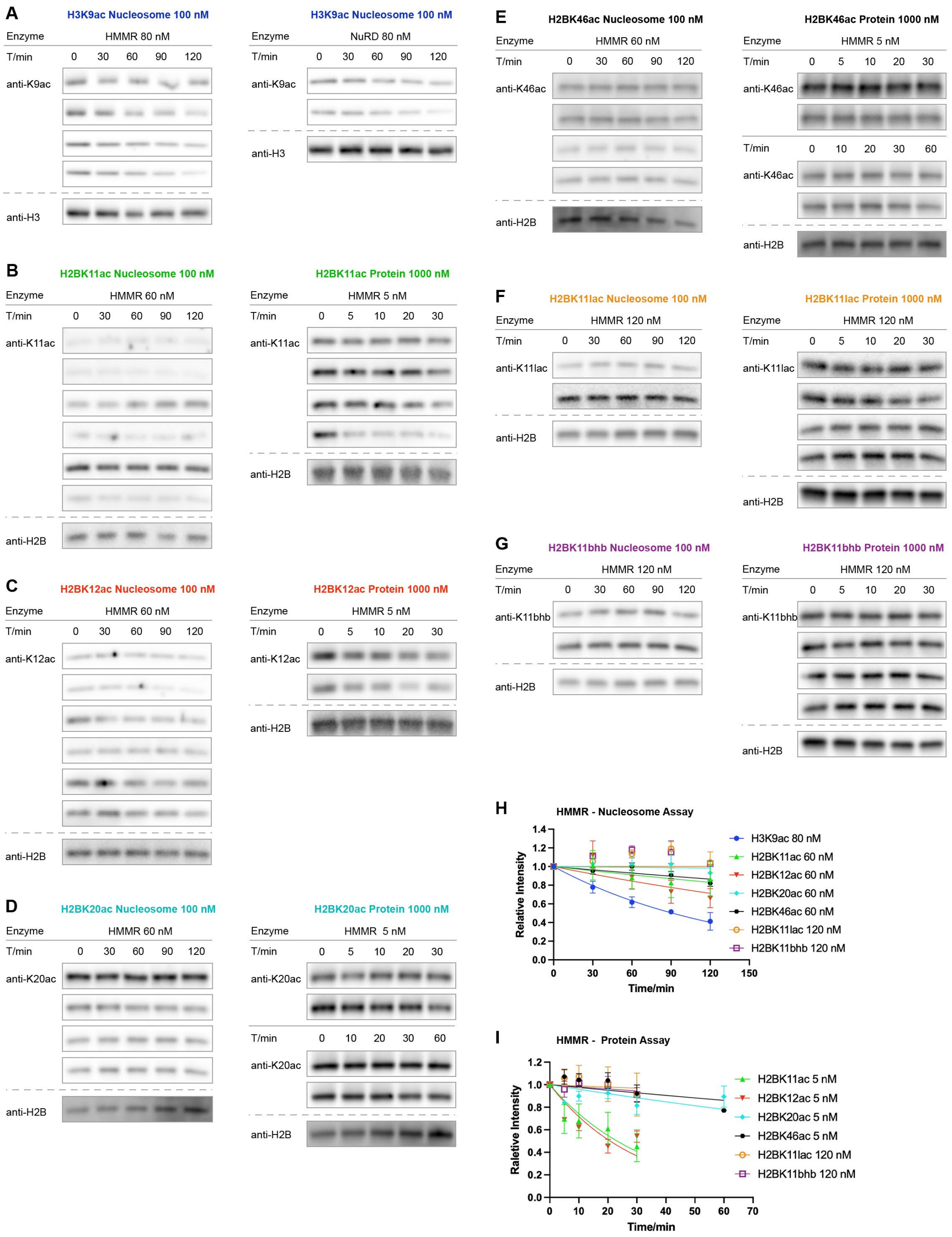

35. Figure S13. WB and curve fitting for deacylation by the HMMR complex

(A) H3K9ac (n = 4 for nucleosome assay with 80 nM HMMR (NuRD bound to MBD2) & n = 2 for nucleosome assay with 80 nM NuRD); (B) H2BK11ac (n = 6 for nucleosome assay; n = 4 for free histone protein assay); (C) H2BK12ac (n = 6 for nucleosome assay; n = 2 for free histone protein assay), (D) H2BK20ac (n = 4 for nucleosome assay; n = 4 for free histone protein assay), (E) H2BK46ac (n = 4 for nucleosome assay; n = 3 for free histone protein assay). (F) H2BK11lac (n = 2 for nucleosome assay; n = 4 for free histone protein assay); (G) H2BK11bhb (n = 2 for nucleosome assay; n = 4 for free histone protein assay); (H) Curve fitting for nucleosome kinetics with 60 or 120 nM HMMR labeled in the graph; (I) Curve fitting for histone kinetics with 5 or 120 nM HMMR labeled in the graph.

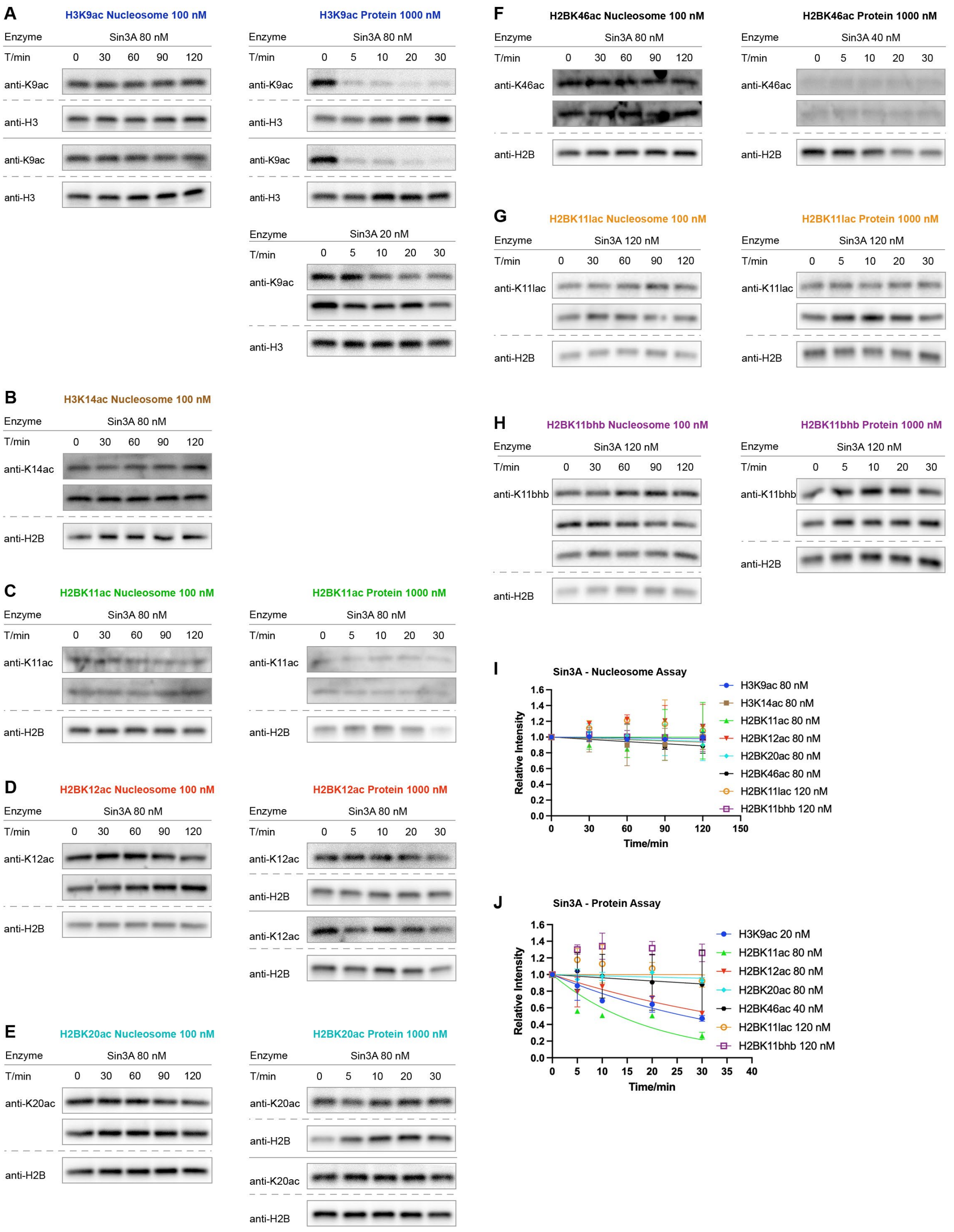

36. Figure S14. WB and curve fitting for deacylation by the Sin3A complex

(A) H3K9ac (n = 2 for nucleosome assay; n = 2 for free histone protein assay with 20 nM Sin3A & n = 2 with 80 nM Sin3A); (B) H3K14ac (n = 2 for nucleosome assay); (C) H2BK11ac (n = 2 for nucleosome assay; n = 2 for free histone protein assay); (D) H2BK12ac (n = 2 for nucleosome assay; n = 2 for free histone protein assay), (E) H2BK20ac (n = 2 for nucleosome assay; n = 2 for free histone protein assay), (F) H2BK46ac (n = 2 for nucleosome assay; n = 2 for free histone protein assay). (G) H2BK11lac (n = 2 for nucleosome assay; n = 2 for free histone protein assay); (H) H2BK11bhb (n = 3 for nucleosome assay; n = 2 for free histone protein assay); (I) Curve fitting for nucleosome kinetics with 80, or 120 nM Sin3A labeled in the graph; (J) Curve fitting for histone kinetics with 20, 40, 80, or 120 nM Sin3A labeled in the graph.

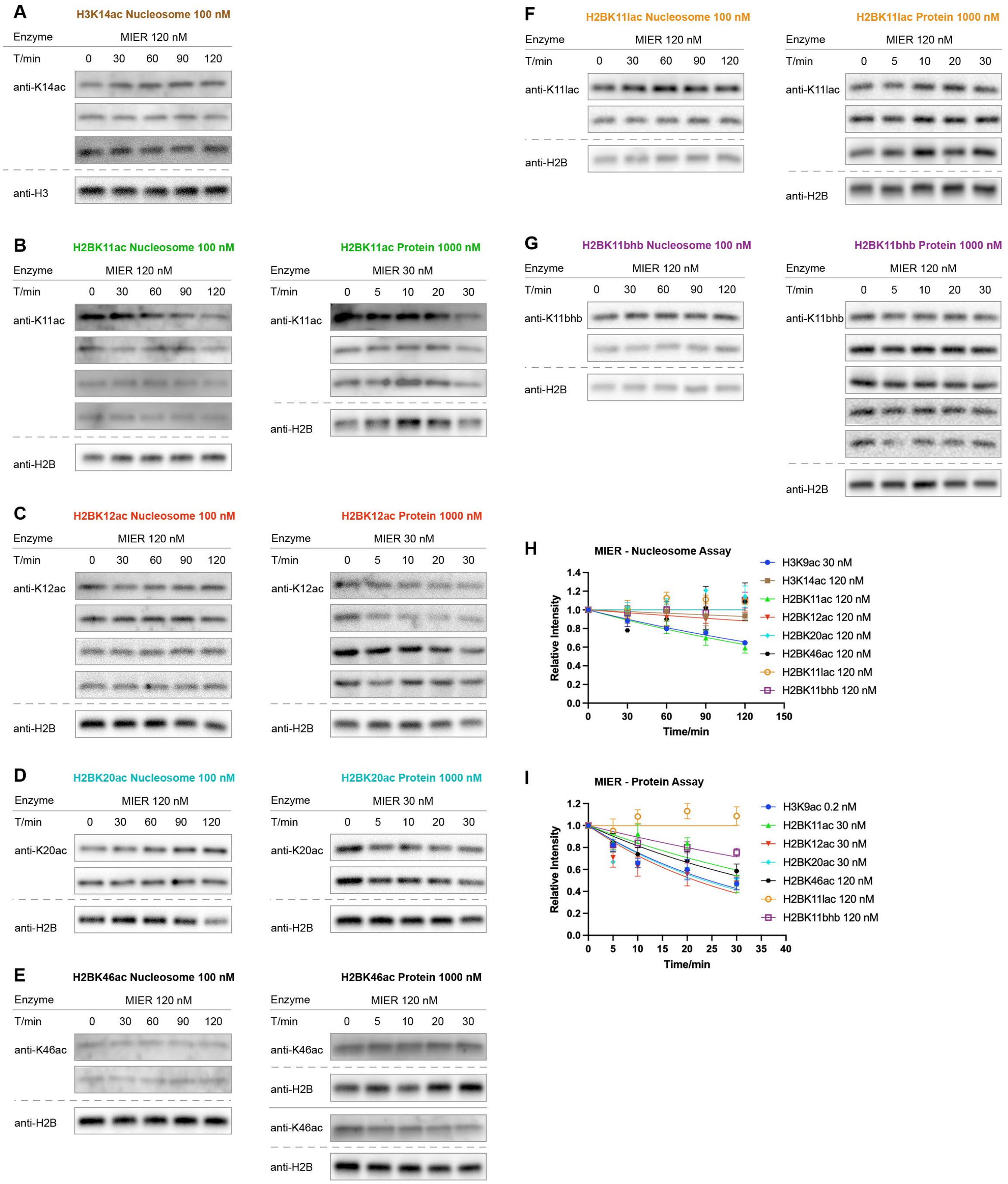

37. Figure S15. WB and curve fitting for deacylation by the MIER complex

(A) H3K14ac (n = 3 for nucleosome assay); (B) H2BK11ac (n = 4 for nucleosome assay; n = 3 for free histone protein assay); (C) H2BK12ac (n = 4 for nucleosome assay; n = 4 for free histone protein assay), (D) H2BK20ac (n = 2 for nucleosome assay; n = 2 for free histone protein assay), (E) H2BK46ac (n = 2 for nucleosome assay; n = 2 for free histone protein assay). (F) H2BK11lac (n = 2 for nucleosome assay; n = 3 for free histone protein assay); (G) H2BK11bhb (n = 2 for nucleosome assay; n = 5 for free histone protein assay); (H) Curve fitting for nucleosome kinetics with 30, or 120 nM MIER labeled in the graph; (I) Curve fitting for histone kinetics with 0.2, 30, or 120 nM MIER labeled in the graph.

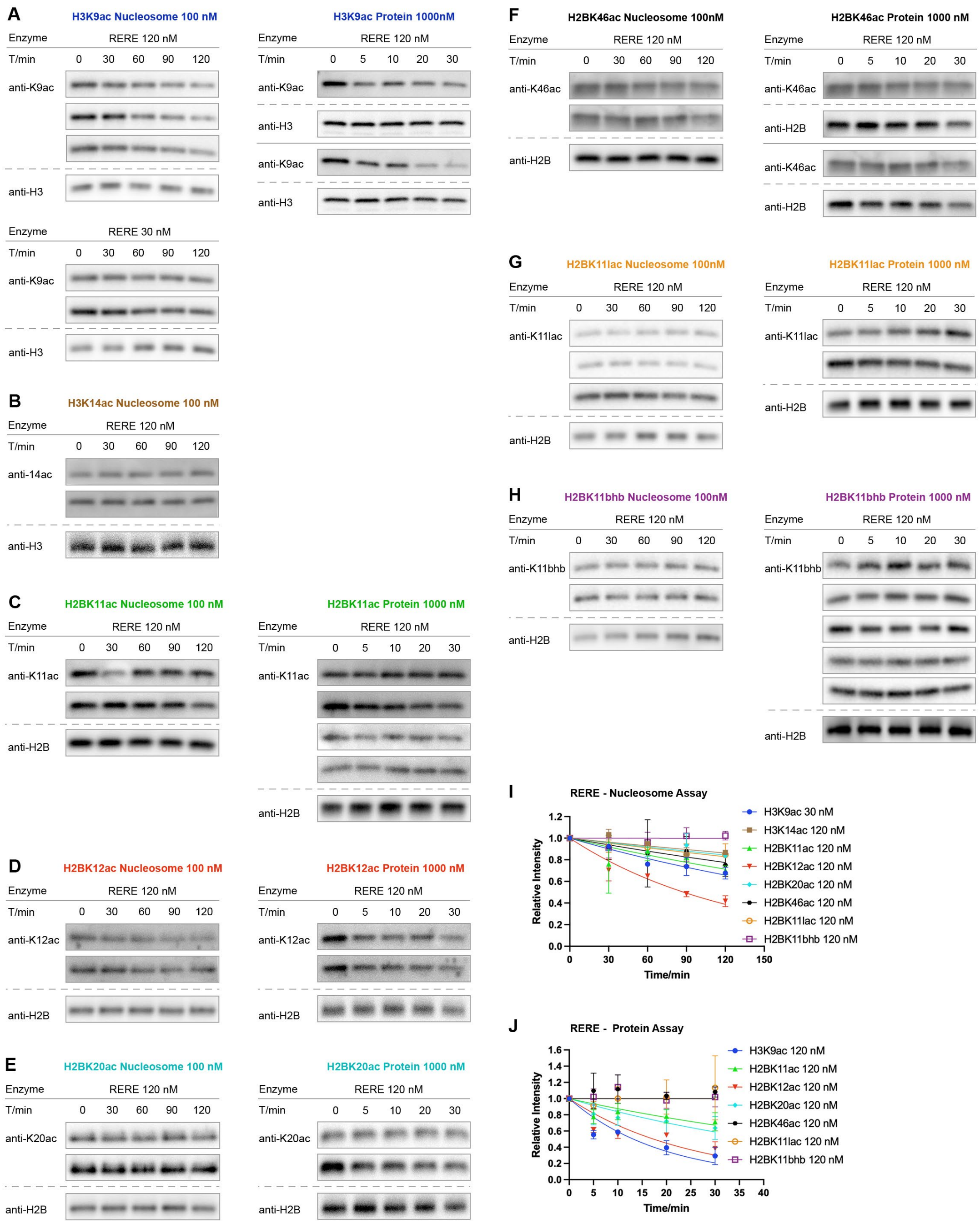

38. Figure S16. WB and curve fitting for deacylation by the RERE complex

(A) H3K9ac (n = 2 for nucleosome assay with 30 nM RERE & n = 3 with 120 nM RERE; n = 2 for free histone protein assay); (B) H3K14ac (n = 2 for nucleosome assay); (C) H2BK11ac (n = 2 for nucleosome assay; n = 4 for free histone protein assay); (D) H2BK12ac (n = 2 for nucleosome assay; n = 2 for free histone protein assay), (E) H2BK20ac (n = 2 for nucleosome assay; n = 2 for free histone protein assay), (F) H2BK46ac (n = 2 for nucleosome assay; n = 2 for free histone protein assay). (G) H2BK11lac (n = 3 for nucleosome assay; n = 2 for free histone protein assay); (H) H2BK11bhb (n = 2 for nucleosome assay; n = 5 for free histone protein assay); (I) Curve fitting for nucleosome kinetics with 30, or 120 nM RERE labeled in the graph; (J) Curve fitting for histone kinetics with 120 nM RERE labeled in the graph.

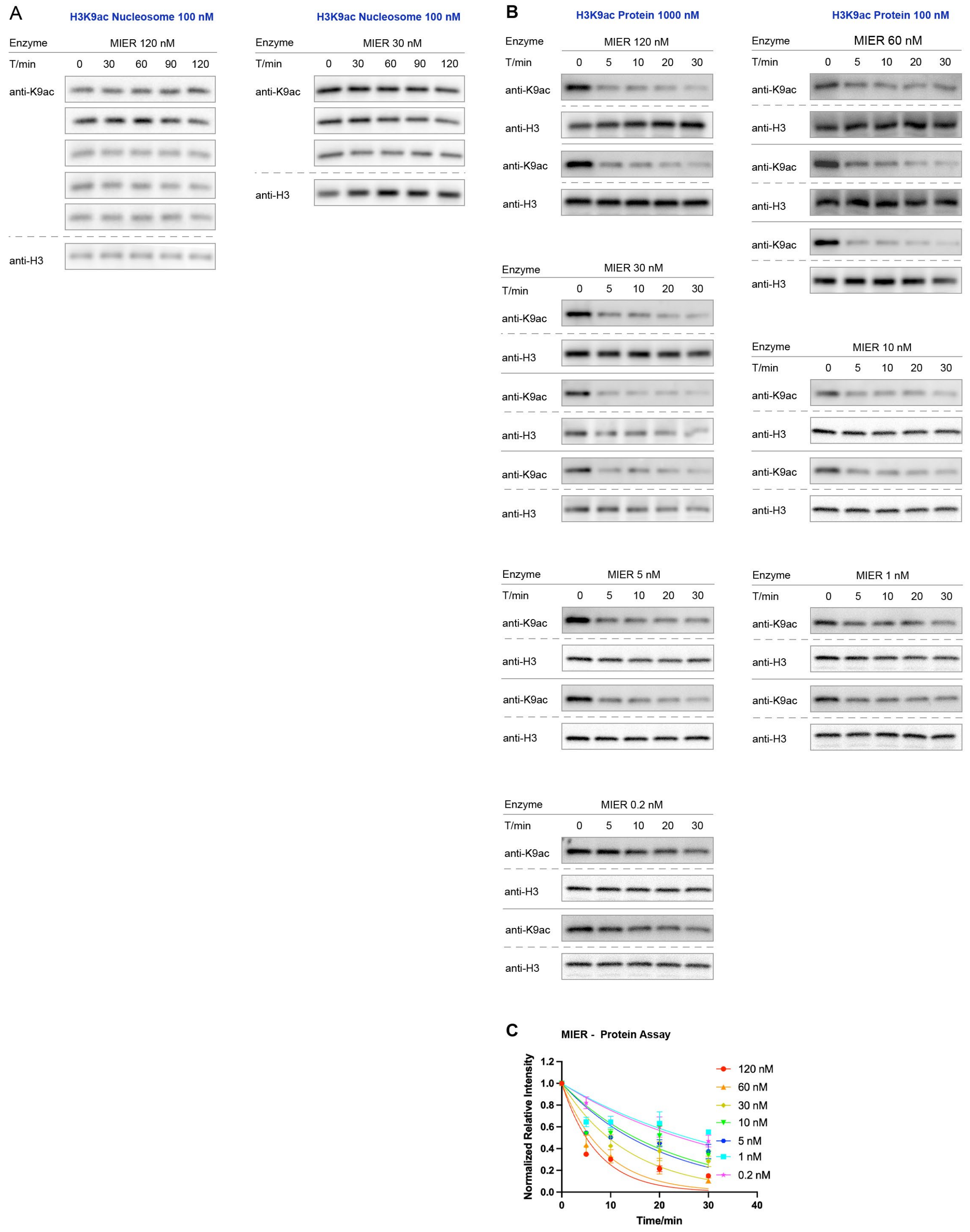

39. Figure S17. Enzyme concentration-dependent assay for MIER

(A) H3K9ac nucleosome (n = 5 with 120 nM MIER & n = 3 with 30 nM MIER) (B) H3K9ac protein (n = 2 with 120 nM MIER, n = 3 with 60 nM MIER, n = 3 with 30 nM MIER, n = 2 with 10 nM MIER, n = 2 with 5 nM MIER, n = 2 with 1 nM MIER, & n = 2 with 0.2 nM MIER).

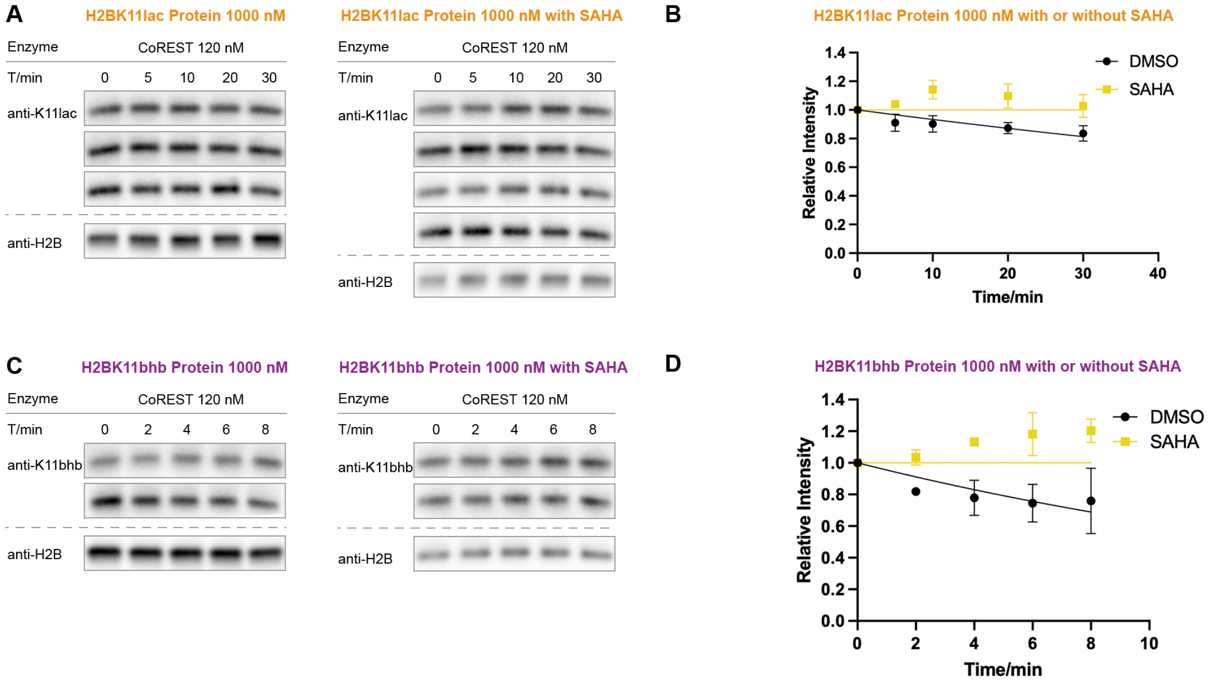

E

Protein	CoREST w/ SAHA	CoREST w/ DMSO
H2BK11lac	<0.001	0.011±0.0024
H2BK11bhb	<0.001	0.078±0.020

40. Figure S18. SAHA inhibition assay for CoREST on H2BK11lac and H2BK11bhb

(A) WB of the CoREST deacylation assay on H2BK11lac protein with or without 10 μM SAHA; (B) Curve-fitting of the CoREST deacylation assay on H2BK11lac protein with or without 10 μM SAHA; (C) WB of the CoREST deacylation assay on H2BK11bhb protein with or without 10 μM SAHA; (D) Curve-fitting of the CoREST deacylation assay on H2BK11bhb protein with or without 10 μM SAHA; (E) Table for V/[E].

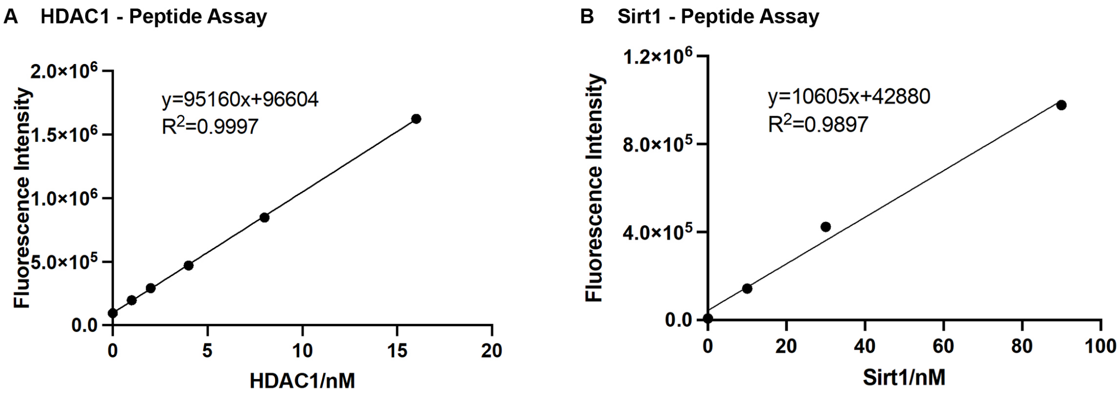

41. Figure S19. Fluor de lys assay for HDAC1 and sirtuin 1

(A) Fluor de lys assay for a gradient of commercial HDAC1 (0, 1 nM, 2 nM, 4 nM, 8 nM, 16 nM) was carried out with fluorescence HDAC1 assay kit; (B) Fluor de lys assay for a gradient of commercial Sirt1 (0, 10 nM, 30 nM, 90 nM) was carried out with fluorescence sirtuin assay kit.

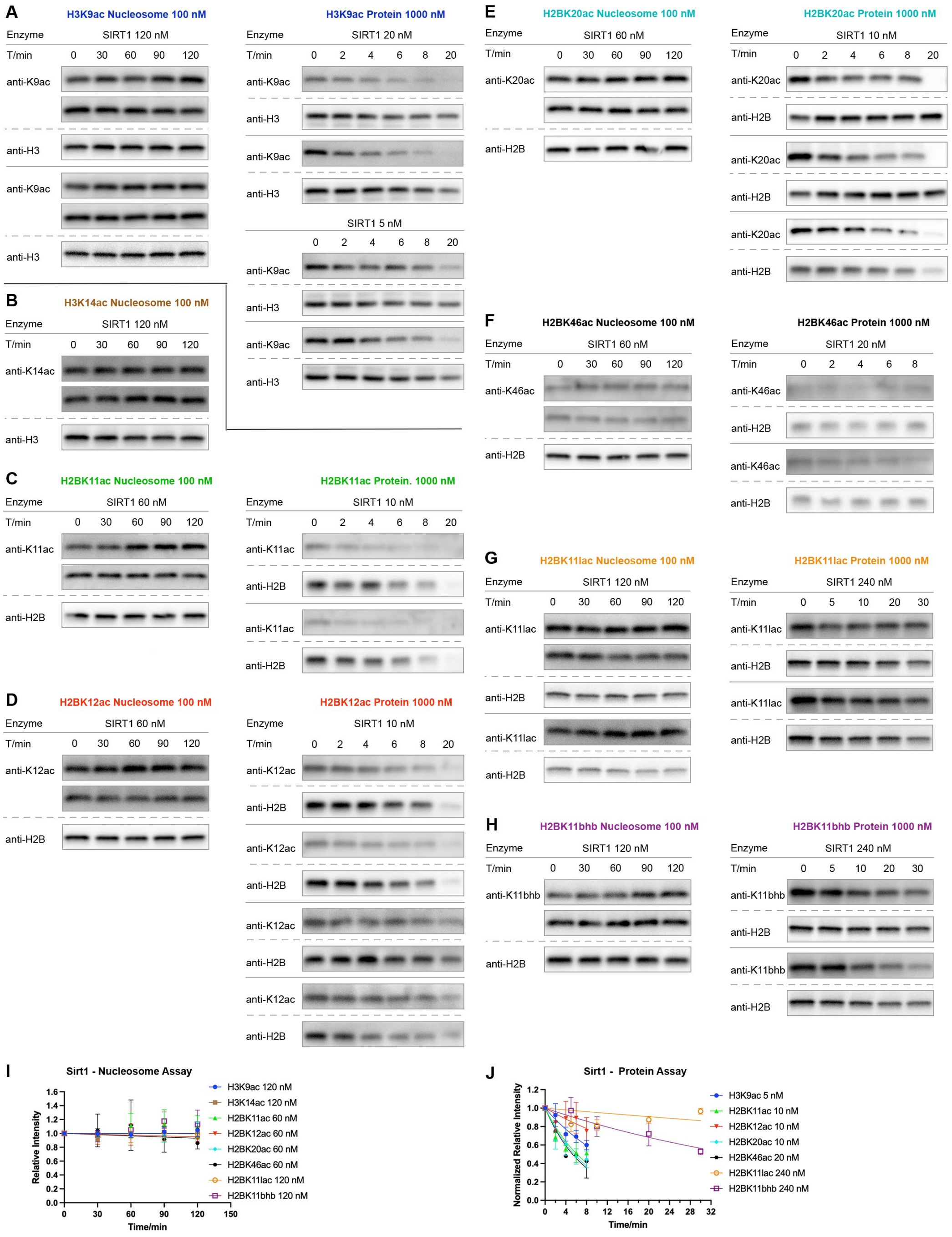

42. Figure S20. WB and curve fitting for deacylation by sirtuin 1

(A) H3K9ac (n = 4 for nucleosome assay; n = 2 for free histone protein assay with 5 nM Sirt1 & n = 2 with 20 nM Sirt1); (B) H3K14ac (n = 2 for nucleosome assay); (C) H2BK11ac (n = 2 for nucleosome assay; n = 2 for free histone protein assay); (D) H2BK12ac (n = 2 for nucleosome assay; n = 4 for free histone protein assay), (E) H2BK20ac (n = 2 for nucleosome assay; n = 3 for free histone protein assay), (F) H2BK46ac (n = 2 for nucleosome assay; n = 2 for free histone protein assay). (G) H2BK11lac (n = 3 for nucleosome assay; n = 2 for free histone protein assay); (H) H2BK11bhb (n = 2 for nucleosome assay; n = 2 for free histone protein assay); (I) Curve fitting for nucleosome kinetics with 60, or 120 nM Sirt1 labeled in the graph; (J) Curve fitting for histone kinetics with 5, 10, 20, or 240 nM Sirt1 labeled in the graph.

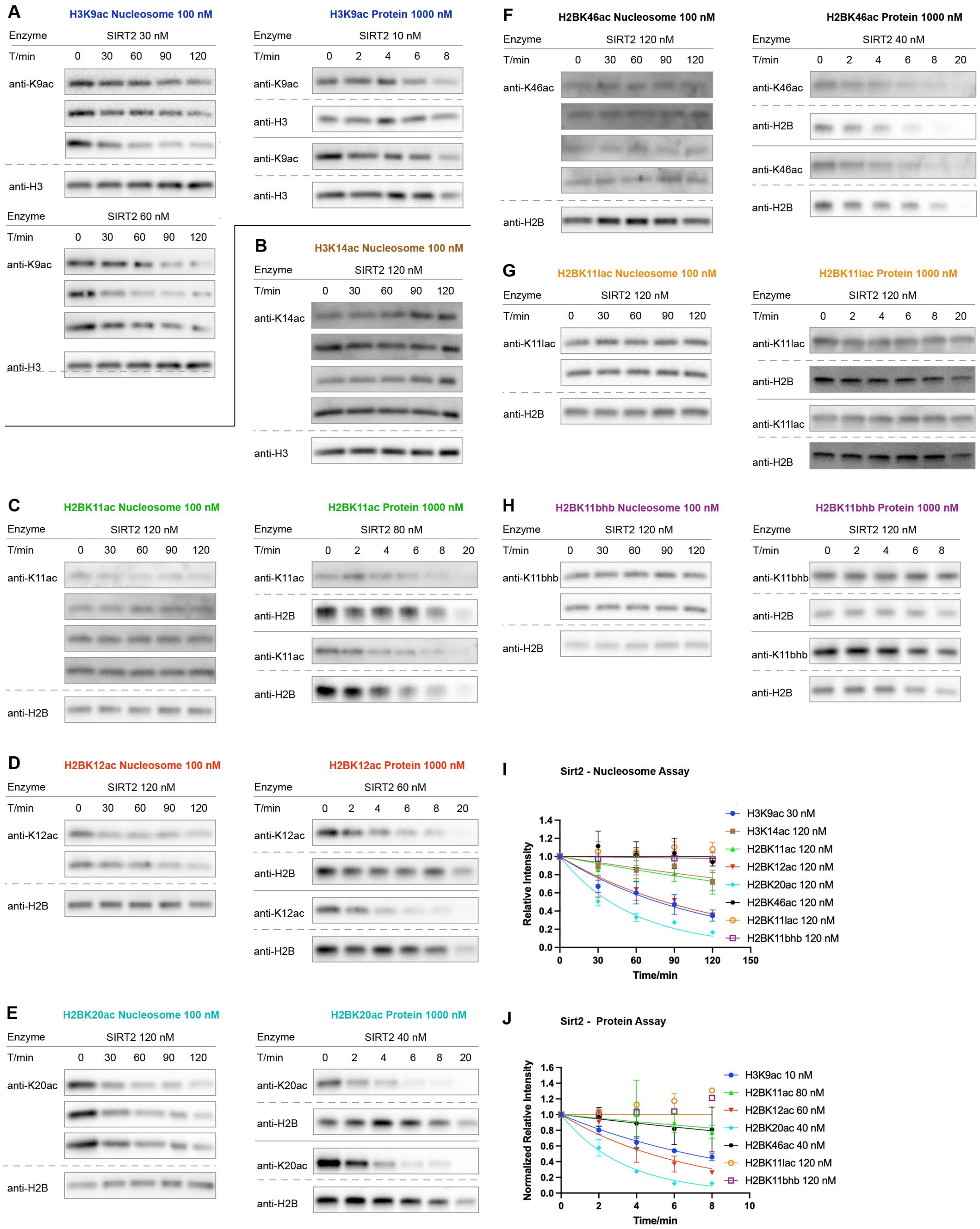

43. Figure S21. WB and curve fitting for deacylation by sirtuin 2

(A) H3K9ac (n = 3 for nucleosome assay with 30 nM Sirt2 & n = 3 with 60 nM Sirt2; n = 2 for free histone protein assay); (B) H3K14ac (n = 4 for nucleosome assay); (C) H2BK11ac (n = 4 for nucleosome assay; n = 2 for free histone protein assay); (D) H2BK12ac (n = 2 for nucleosome assay; n = 2 for free histone protein assay), (E) H2BK20ac (n = 3 for nucleosome assay; n = 2 for free histone protein assay), (F) H2BK46ac (n = 4 for nucleosome assay; n = 2 for free histone protein assay). (G) H2BK11lac (n = 2 for nucleosome assay; n = 2 for free histone protein assay); (H) H2BK11bhb (n = 2 for nucleosome assay; n = 2 for free histone protein assay); (I) Curve fitting for nucleosome kinetics with 30, or 120 nM Sirt2 labeled in the graph; (J) Curve fitting for histone kinetics with 10, 40, 60, 80, or 120 nM Sirt2 labeled in the graph.

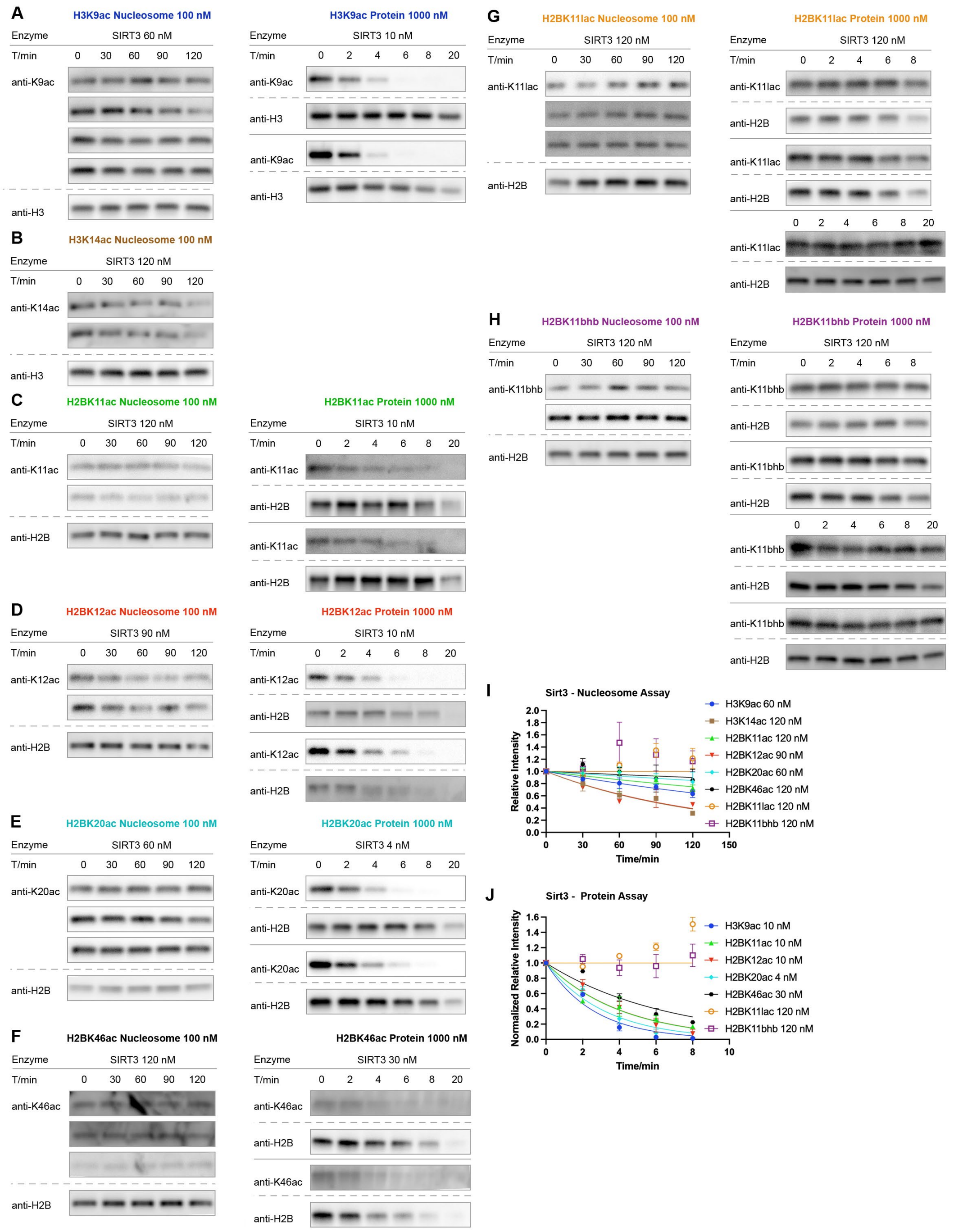

44. Figure S22. WB and curve fitting for deacylation by sirtuin 3

(A) H3K9ac (n = 4 for nucleosome assay; n = 2 for free histone protein assay); (B) H3K14ac (n = 2 for nucleosome assay); (C) H2BK11ac (n = 2 for nucleosome assay; n = 2 for free histone protein assay); (D) H2BK12ac (n = 2 for nucleosome assay; n = 2 for free histone protein assay), (E) H2BK20ac (n = 3 for nucleosome assay; n = 2 for free histone protein assay), (F) H2BK46ac (n = 3 for nucleosome assay; n = 2 for free histone protein assay). (G) H2BK11lac (n = 3 for nucleosome assay; n = 3 for free histone protein assay); (H) H2BK11bhb (n = 2 for nucleosome assay; n = 4 for free histone protein assay); (I) Curve fitting for nucleosome kinetics with 60, or 120 nM Sirt3 labeled in the graph; (J) Curve fitting for histone kinetics with 4, 10, 30, or 120 nM Sirt3 labeled in the graph.

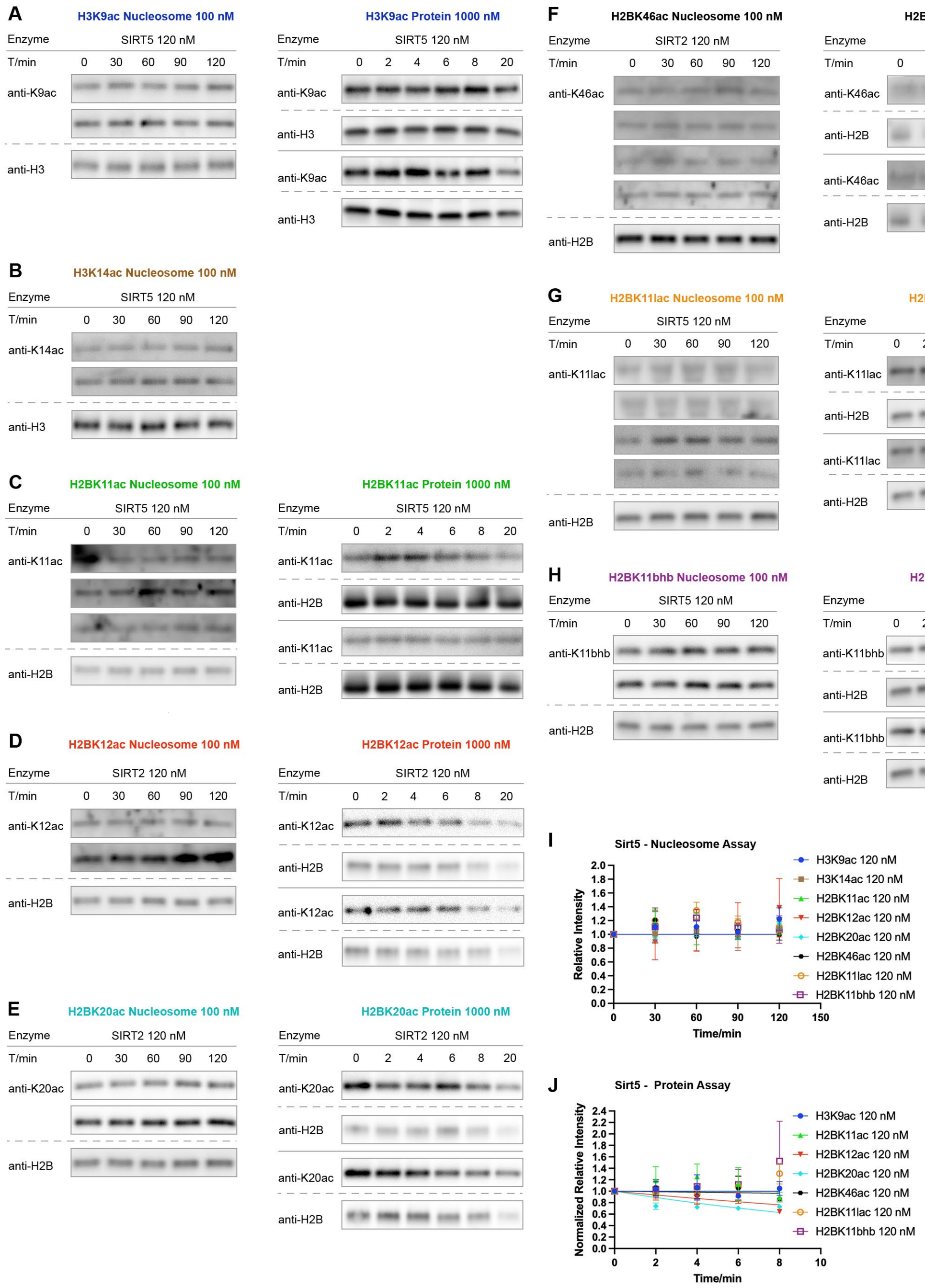

45. Figure S23. WB and curve fitting for deacylation by sirtuin 5

(A) H3K9ac (n = 2 for nucleosome assay; n = 2 for free histone protein assay); (B) H3K14ac (n = 2 for nucleosome assay); (C) H2BK11ac (n = 3 for nucleosome assay; n = 2 for free histone protein assay); (D) H2BK12ac (n = 2 for nucleosome assay; n = 2 for free histone protein assay), (E) H2BK20ac (n = 2 for nucleosome assay; n = 2 for free histone protein assay), (F) H2BK46ac (n = 4 for nucleosome assay; n = 2 for free histone protein assay). (G) H2BK11lac (n = 4 for nucleosome assay; n = 2 for free histone protein assay); (H) H2BK11bhb (n = 2 for nucleosome assay; n = 2 for free histone protein assay); (I) Curve fitting for nucleosome kinetics with or 120 nM Sirt5 labeled in the graph; (J) Curve fitting for histone kinetics with 120 nM Sirt5 labeled in the graph.

46. Table S2. V/[E] of all HDAC1 complexes and sirtuins on H2B and H3 nucleosomes

Nucleosome	HDAC1	CoREST	MiDAC	HMMR	Sin3A	MIER	RERE	Sirt1	Sirt2	Sirt3	Sirt5
H3K9ac	< 0.005*	0.14±0.0056	1.1±0.025	0.0095±0.00081	< 0.001	0.012±0.00077	0.012±0.0011	< 0.001	0.030±0.0036	0.0060±0.00071	< 0.001
H3K14ac	< 0.005*	< 0.005*	0.52*	0.0091*	< 0.001	< 0.001	0.0010±0.00022	< 0.001	0.0019±0.00041	0.0067±0.00063	< 0.001
H2BK11ac	< 0.001	0.032±0.0029	2.4±0.26	0.0026±0.0016	< 0.001	0.0032±0.00041	0.0023±0.00075	< 0.001	0.0022±0.00061	0.0020±0.00055	< 0.001
H2BK12ac	< 0.001	0.026±0.0030	0.84±0.048	0.0047±0.0015	< 0.001	< 0.001	0.0066±0.00053	< 0.001	0.0070±0.00078	0.0087±0.0010	< 0.001
H2BK20ac	< 0.001	0.014±0.0011	0.0022±0.00056	< 0.001	< 0.001	< 0.001	0.0012±0.00029	< 0.001	0.015±0.00097	0.0022±0.0010	< 0.001
H2BK46ac	< 0.001	0.0076±0.0018	< 0.001	0.0020±0.00040	< 0.001	< 0.001	0.0018±0.00079	< 0.001	< 0.001	< 0.001	< 0.001
H2BK11lac	< 0.001	< 0.001	0.0034±0.00034	< 0.001	< 0.001	< 0.001	0.0013±0.00038	< 0.001	< 0.001	< 0.001	< 0.001
H2BK11bhb	< 0.001	< 0.001	0.0043±0.00063	< 0.001	< 0.001	< 0.001	< 0.001	< 0.001	< 0.001	< 0.001	< 0.001

All data are rounded to two significant figures. * indicates data reported previously.[8] < 0.001 reflects undetectable deacetylation.

47. Table S3. Normalized V/[E] of all HDAC1 complexes and sirtuins on H2B and H3 free proteins

Protein	HDAC1	CoREST	MiDAC	HMMR	Sin3A	MIER	RERE	Sirt1	Sirt2	Sirt3	Sirt5
H3K9ac	0.30*	2.1*	0.42*	0.84*	0.26±0.037	28±3.0	0.088±0.013	2.6±0.34	2.1±0.13	7.6±0.75	< 0.02
H2BK11ac	1.0±0.14	3.6±0.52	0.099±0.011	1.2±0.26	0.13±0.020	0.12±0.021	0.022±0.0053	2.3±0.26	0.059±0.046	4.8±0.47	< 0.02
H2BK12ac	0.73±0.087	4.3±0.70	0.030±0.0065	1.3±0.21	0.049±0.0080	0.21±0.031	0.067±0.011	0.63±0.28	0.55±0.075	4.8±0.49	0.057±0.018
H2BK20ac	0.10±0.0081	0.073±0.0085	0.017±0.0029	0.16±0.048	< 0.02	0.20±0.029	0.029±0.0067	2.5±0.33	1.5±0.11	16±1.5	0.097±0.015
H2BK46ac	0.29±0.039	0.083±0.012	0.023±0.0066	0.10±0.045	< 0.02	0.034±0.0045	< 0.02	1.3±0.17	0.14±0.091	1.0±0.12	< 0.02
H2BK11lac	< 0.02	0.023±0.0024	< 0.02	< 0.02	< 0.02	< 0.02	< 0.02	< 0.02	< 0.02	< 0.02	< 0.02
H2BK11bhb	< 0.02	0.030±0.0052	< 0.02	< 0.02	< 0.02	0.019±0.0024	< 0.02	0.016±0.0028	< 0.02	< 0.02	< 0.02

All data are rounded to two significant figures. * indicates data reported previously.[8] < 0.02 reflects undetectable deacetylation. Normalized V/[E] were calculated (V/[E] divided by a factor of 5) given that each nucleosome contains two H3 molecules and the final histone protein substrate (1.0 μM) is ten times more than nucleosome substrate (100 nM).

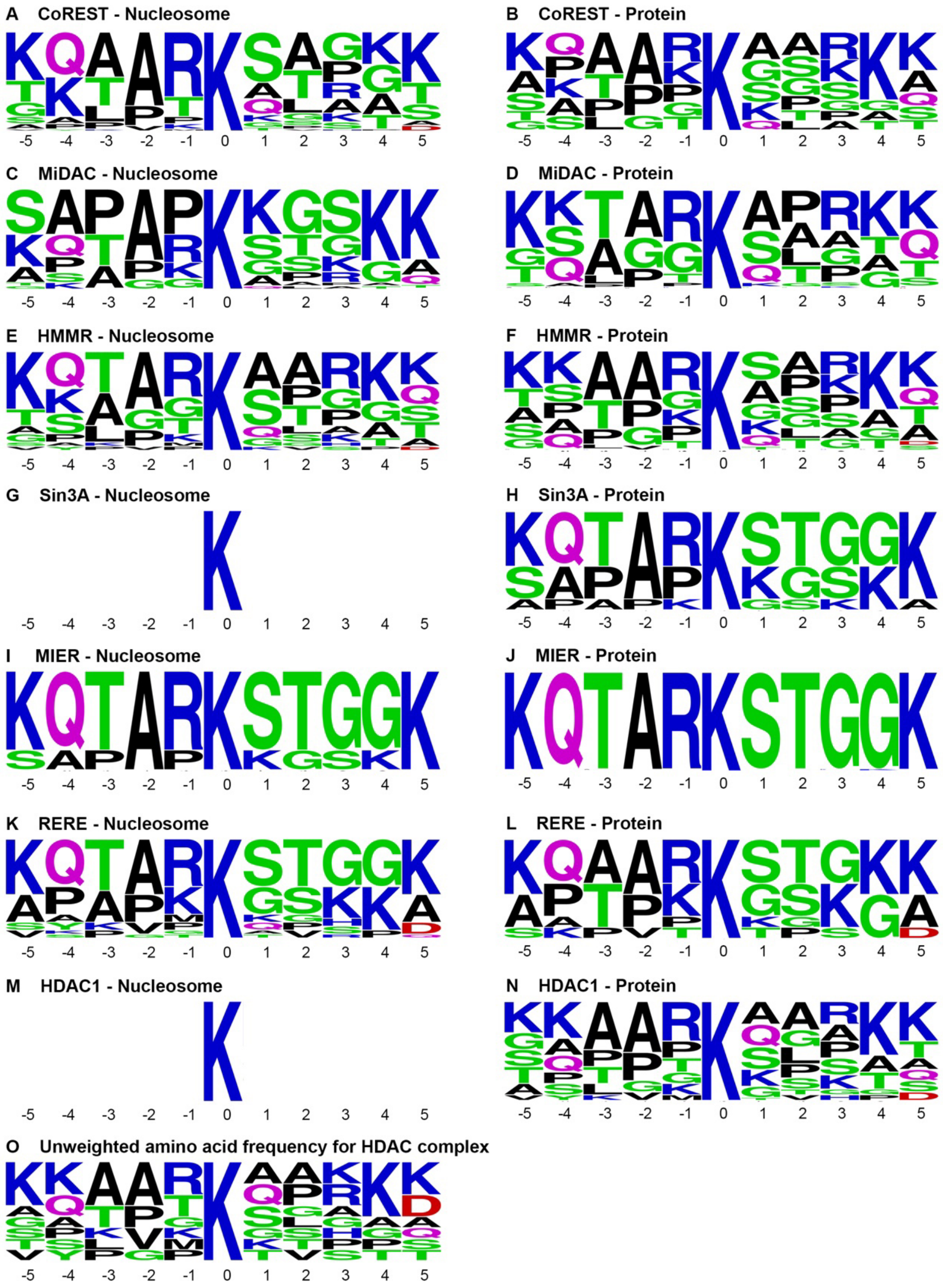

48. Figure S24. Rate-weighted sequence features of HDAC complex target sites

(A) CoREST on nucleosome; (B) CoREST on protein. (C) MiDAC on nucleosome; (D) MiDAC on protein. (E) HMMR on nucleosome; (F) HMMR on protein. (G) Sin3A on nucleosome; (H) Sin3A on protein. (I) MIER on nucleosome; (J) MIER on protein. (K) RERE on nucleosome; (L) RERE on protein. (M) HDAC1 on nucleosome; (N) HDAC1 on protein; (O) unweighted amino acid frequencies surrounding the acetylation sites studied with HDAC and its complexes.

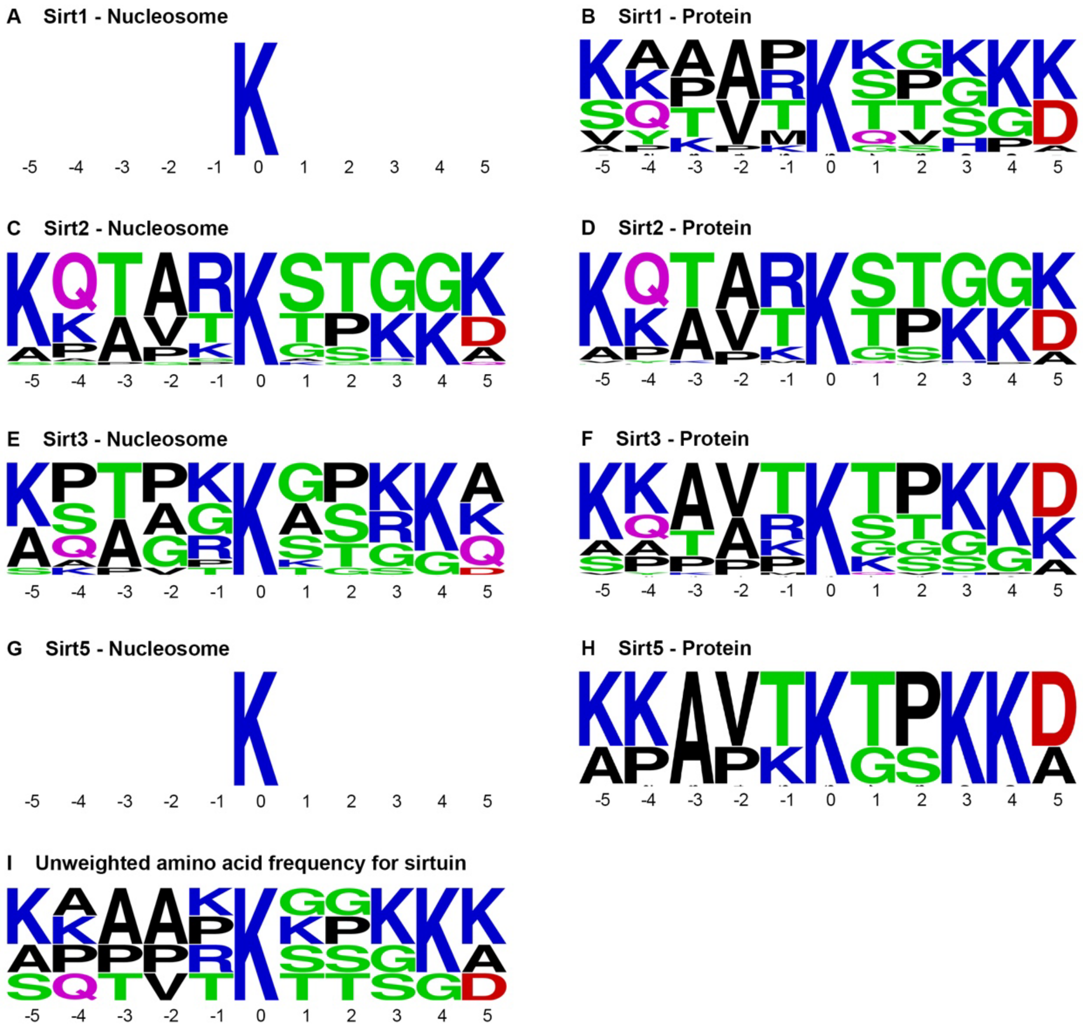

49. Figure S25. Rate-weighted sequence features of sirtuin target sites

(A) Sirt1 on nucleosome; (B) Sirt1 on protein. (C) Sirt2 on nucleosome; (D) Sirt2 on protein. (E) Sirt3 on nucleosome; (F) Sirt3 on protein. (G) Sirt5 on nucleosome; (H) Sirt5 on protein; (I) unweighted amino acid frequencies surrounding the acetylation sites studied with Sirtuins.

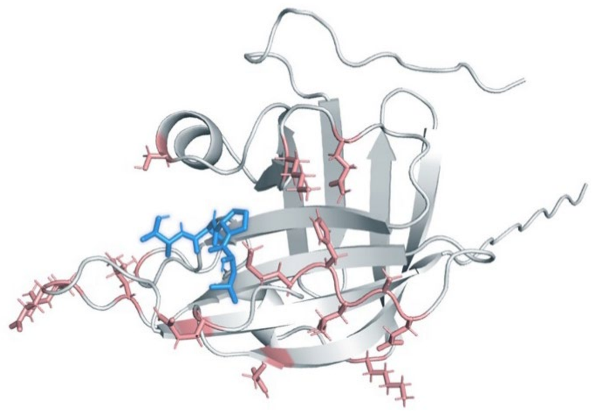

50. Figure S26. W4 sortase mutation sites

An existing solution structure (PDBID: 2KID) of sortase A[18] (grey) in complex with a substrate analogue (blue) was used to model W4 mutations. Amino acid substitutions (pink) were introduced using PyMol.[19] The mutant structure, excluding the substrate analogue, was refined using GalaxyRefine2.[20]
